# Kinetic Validation of the Models for P-Glycoprotein ATP Hydrolysis and Vanadate-Induced Trapping. Proposal for Additional Steps

**DOI:** 10.1371/journal.pone.0098804

**Published:** 2014-06-04

**Authors:** Miguel Ramón Lugo, Frances Jane Sharom

**Affiliations:** Department of Molecular and Cellular Biology, University of Guelph, Guelph, Ontario, Canada; University of Technology Sydney, Australia

## Abstract

P-Glycoprotein, a member of the ATP-binding cassette (ABC) superfamily, is a multidrug transporter responsible for cellular efflux of hundreds of structurally unrelated compounds, including natural products, many clinically used drugs and anti-cancer agents. Expression of P-glycoprotein has been linked to multidrug resistance in human cancers. ABC transporters are driven by ATP hydrolysis at their two cytoplasmic nucleotide-binding domains, which interact to form a closed ATP-bound sandwich dimer. Intimate knowledge of the catalytic cycle of these proteins is clearly essential for understanding their mechanism of action. P-Glycoprotein has been proposed to hydrolyse ATP by an alternating mechanism, for which there is substantial experimental evidence, including inhibition of catalytic activity by trapping of *ortho*-vanadate at one nucleotide-binding domain, and the observation of an asymmetric occluded state. Despite many studies of P-glycoprotein ATPase activity over the past 20 years, no comprehensive kinetic analysis has yet been carried out, and some puzzling features of its behaviour remain unexplained. In this work, we have built several progressively more complex kinetic models, and then carried out simulations and detailed analysis, to test the validity of the proposed reaction pathway employed by P-glycoprotein for ATP hydrolysis. To establish kinetic parameters for the catalytic cycle, we made use of the large amount of published data on ATP hydrolysis by hamster P-glycoprotein, both purified and in membrane vesicles. The proposed kinetic scheme(s) include a high affinity priming reaction for binding of the first ATP molecule, and an independent pathway for ADP binding outside the main catalytic cycle. They can reproduce to varying degrees the observed behavior of the protein's ATPase activity and its inhibition by *ortho*-vanadate. The results provide new insights into the mode of action of P-glycoprotein, and some hypotheses about the nature of the occluded nucleotide-bound state.

## Introduction

The multidrug transporter P-glycoprotein (Pgp, ABCB1) is a plasma membrane protein belonging to the ABC superfamily which couples the efflux of a wide variety of chemically and structurally different compounds to the hydrolysis of ATP [1]. Commonly used chemotherapy drugs are transported by Pgp, and its overexpression in tumour cells is linked to the multidrug resistant (MDR) phenotype that many human cancers present in the clinic [2], [3]. Following the first report of Pgp ATPase activity [4], studies characterizing ATP hydrolysis were conducted in the early 1990s with plasma membrane preparations from MDR cell-lines [5], partially purified [6], [7] or purified detergent-solubilized Pgp [8], [9], and reconstituted Pgp [10], [11]. Since then, the catalytic cycle of the enzyme, its coupling to drug transport, and its inhibition by *ortho*-vanadate (V_i_) have been studied by several research groups [12], [13].

In 1995, Senior's group published a minimal reaction pathway for hydrolysis of one molecule of ATP by Pgp, and V_i_-induced inhibition of its catalytic activity [14]. The protein possesses two consensus sequences for ATP binding, located within the two nucleotide binding domains (NBD1 and NBD2) in the highly homologous halves. In support of the proposed scheme for the catalytic reaction, it was demonstrated that both NBD1 and NBD2 are capable of binding and hydrolysing ATP [14]–[16]. Thus, the minimal reaction scheme presented for the hydrolysis of ATP and trapping by V_i_ corresponds to the catalytic activity carried out independently by each half-molecule. Consequently, the apparent single *K_m_* observed for ATP hydrolysis [5], [9], and the apparent single *K_d_* reported for binding of nucleotides and nucleotide analogs observed by fluorescence and EPR spectroscopy [17]–[20], suggest that NBD1 and NBD2 are similar in their binding and kinetic properties in regard to the hydrolysis of ATP.

It is now generally accepted that the two NBDs of ABC proteins must interact to form a sandwich dimer for the normal functioning of these proteins, and such cooperativity has been shown for Pgp [21]. Thus, inactivation of one of the catalytic sites by either mutation [22] or chemical modification [15], or the formation of a non-covalent long-lived complex with V_i_ trapped at a single NBD [14], [23], is enough to completely abolish the ATPase activity of the enzyme. As result, steady-state catalysis takes place only when both half-molecules are intact. In addition, stimulation of the basal ATP activity by drug substrates is displayed only when the full-length transporter is expressed, or both half-molecules are co-expressed [24].

Based on a minimal reaction scheme, Senior and co-workers [25] were the first to postulate a model for coupling between the catalytic activities of the two NBDs, under the name *Alternating Catalytic Mechanism*. In this model, the hydrolytic reactions of each half-cycle, and the accompanying protein conformational changes, alternate to carry out the transport of a drug molecule. The catalytic activity at NBD1 containing a bound ATP molecule is triggered when a second ATP molecule binds to NBD2, and vice versa. Thus, the reaction progresses in an alternating sequence of ATP binding and hydrolysis in the complementary half-molecules. In this model, the authors hypothesized that the transport of the drug is coupled to the relaxation of the protein from a high chemical potential state that is generated by the hydrolytic step. In one turnover of this cycle, two molecules of drug are transported and two molecules of ATP are consumed. This basic mechanism for the catalytic and transport cycle of the Pgp is currently widely accepted, with the addition of further adaptations based on structural and energetic considerations.

The *Alternating Catalytic Mechanism* suggests that asymmetry in the two halves of Pgp must be maintained throughout the catalytic cycle, in order to retain the memory of which NBD recently hydrolysed ATP (for a detailed discussion of the role of asymmetry in ABC protein function, see [26]). Using Pgp carrying a mutation in an essential catalytic residue in both NBDs (E552A/E1197A), Tombline et al. demonstrated the existence of a stable asymmetric nucleotide-bound Pgp species [27]. After gel filtration chromatography the protein retained one molecule of ATP, which was bound with 50-fold higher affinity (*K_d_* = 9 µM) compared to wild-type Pgp (*K_d_* = 0.5 mM). This asymmetric species with a single tightly-bound ATP molecule was referred to as the occluded state. Sauna and co-workers reported the occlusion by wild-type Pgp of a single molecule of ATPγS, a very slowly hydrolysable nucleotide analog [28]. More recently, our laboratory reported that wild-type Pgp binds ATPγS to form an asymmetric species with one tightly bound (occluded) nucleotide (*K_d_* = 6 µM) and one loosely-bound nucleotide (*K_d_* = 0.7 mM) [29]. The asymmetric intermediate was proposed to exist transiently during the catalytic cycle, with the occluded nucleotide normally undergoing rapid hydrolysis [27], [29].

In spite of the great advances in understanding the ABC superfamily that have taken place over the last 15 years, no comprehensive kinetic analysis has been carried out to date. The compact mode of catalysis proposed by Senior's group in 1995 has been used to establish possible transport mechanisms (e.g. it was used in the *Sequential Mechanism* proposed by Sauna and Ambudkar [30]), regardless of the fact that several puzzling experimental observations have only been described superficially, and no satisfactory explanation has yet been proposed for them. These previously ignored observations could possibly be key pieces of information in the development of a comprehensive kinetic model for the catalytic cycle of Pgp. In this work, we built several progressively more complex kinetic models, and then carried out simulations and detailed analysis to test their validity in the proposed reaction pathway for the Pgp-mediated hydrolysis of ATP and its inhibition by V_i_. To establish kinetic parameters for the catalytic cycle, we made use of the large amount of accumulated data on verapamil-stimulated ATP hydrolysis by hamster Pgp, both purified and in membrane vesicles. We show that the proposed kinetic scheme(s), which include additional steps, can reproduce to varying degrees the observed behavior of the protein's ATPase activity and its inhibition by V_i_. The results provide new insights into the mode of action of Pgp, and some hypotheses about the nature of the occluded state.

## Methods

### Construction of the Elemental Cycle Kinetic Model

**Figure 1:**
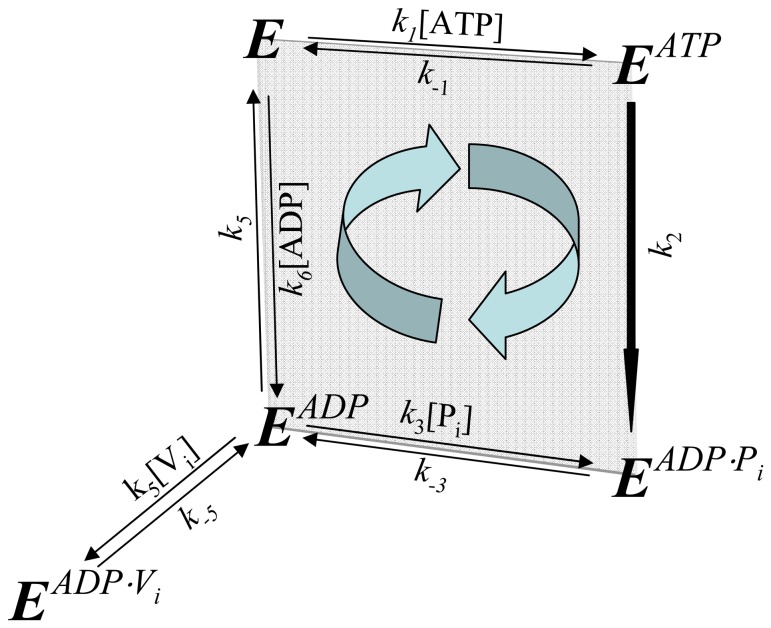
The *Elemental Catalytic Cycle* of Pgp and V_i_-induced inhibition. This scheme for the basic catalytic reaction for ATP hydrolysis by Pgp is adapted from Urbatsch et al. [14] *E*: Pgp.

The basic kinetic cycle consists of an adaptation of the one originally proposed by Senior and co-workers in 1995 [14], [25], here called the *Elemental (Catalytic) Cycle* (**Figure 1**). In it, a single reaction for binding and hydrolysis of MgATP is followed by sequential release of the products P_i_ and MgADP. For brevity, MgATP and MgADP will henceforth be referred to simply as ATP and ADP. Inhibition by V_i_ is achieved by formation of a long-lived complex, with ADP·V_i_ trapped in one catalytic site, by a single step. This complex is thought to resemble structurally the normal transition state conformation formed with P_i_. In our implementation, all the reaction steps were considered reversible except for the ATP hydrolytic step, which is irreversible [31].

The strategy used to test the validity of the reaction scheme in the **Figure 1** consisted of the development of a kinetic model based on the rate law for each reaction. For modeling the scheme in the *Elemental Cycle*, the system was considered as a reaction medium without compartmentalization (which is the case for *in vitro* assays using solubilized enzyme or membrane vesicles) composed of the enzyme (E, Pgp) and one or more ligands (substrate, ATP; inhibitor, V_i_; and products, ADP and P_i_), for a total of n = 5 enzymatic intermediates. From the scheme in **Figure 1**, we formulated a set of ordinary differential equations for the rate of change of the concentration of n-1 intermediates; considering each reaction as an elementary mechanistic step. The reaction system was defined by a vector **C_o_** of initial reactant concentrations, the total concentration of enzyme, [*E*]*_t_*, and a vector **k** constituted of unimolecular and bimolecular rate constants, according to each unidirectional reaction, given by
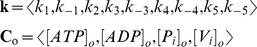
with 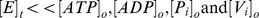. The two following biochemical variables were solved either symbolically or numerically:

- Turnover rate (in s^−1^), *v*

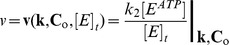

- Fraction of trapped enzyme (adimensional), *T*

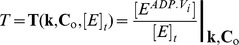

**Figure 2:**
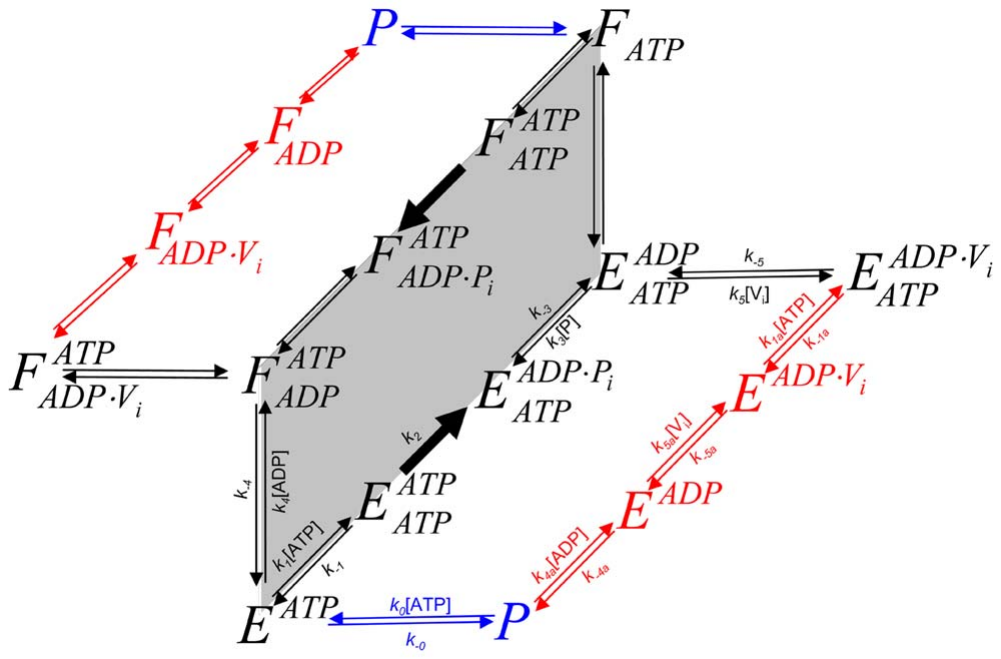
Alternating cycles for Pgp. Scheme based on the original proposal of Senior et al. [25] that includes the coupling of two *Elemental Cycles* of ATP hydrolysis, the trapping reactions with V_i_, the priming reactions with ATP, the priming reactions with ADP, the ADP-dependent V_i_ trapping reactions, and the interconnecting reactions between the ATP and ADP trapping pathways. *E* and *F* represent two ligand-bound isoforms of Pgp (*P*, the bare enzyme) with the ability to hydrolyze ATP in NBD1 (superscript position) and NBD2 (subscript position), respectively. The nomenclature for the rate constants corresponds to that defined for the *Elemental Cycle* (**Figure 1**, rate constants in **Table 2**). The cycle (shaded area) supplemented with the blue reactions corresponds to the *PE Alternating Cycle* (rate constants in **Table 3**). The addition of the red reactions defines the *Extended Alternating Cycle* (rate constants in **Table 4**).

For modeling the other reaction schemes derived from the *Elemental Cycle*, in what are called extensions of the *Alternating Cycle*, additional differential equations were included to account for the new intermediates. In this regard, for the *Partial-Extended Alternating Cycle* (**Figure 2**, including blue reactions) the variables *v* and *T* are defined by the expressions
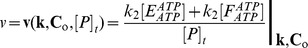


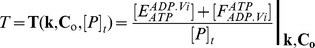
with *P*, *E* and *F* defined in **Figure 2**, and the vector **k** upgraded to include 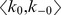. Furthermore, additional reaction paths were added to account for the *Extended Alternating Cycle* (**Figure 2**, including red reactions), for which the following new variables were defined: the fraction of single-nucleotide trapped species, *T*_I_, and the fraction of two-nucleotide trapped species, *T*_II_, given by
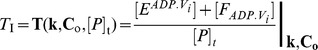


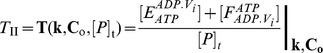
by upgrading the vector **k** to include 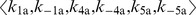.

The analytical solutions were obtained using the computational algebra package GROEBNER included in Maple 15 (MapleSoft Inc., Waterloo ON, Canada), while the general-purpose simulation package SCoP 3.5 (Simulation Resources Inc., MI, USA) was used for numerical integration. For some plotting and fitting procedures, OriginPro 8 was used (OriginLab Corp. MA, USA).

The solutions to these functions were used to kinetically evaluate the self-consistency of the models in accounting for all the experimental evidence, using as a biochemical system hamster Pgp in the presence of the substrate verapamil, in either native plasma membrane, or purified and solubilized in detergent. **Table 1** presents a summary of the values reported for hamster Pgp from the main papers in the field, including (i) phenomenological parameters such as *IC*_50_, 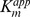, 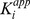, *T_max_*, *v_max_* in steady-state kinetic experiments, or *k_obs_* in time-course experiments, from both catalytic activity and trapping experiments; and (ii) thermodynamic and kinetic parameters such as 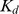, 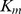, 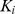 and 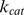.

**Table 1:** Phenomenological and thermodynamic parameters for the ATPase activity and V_i_-induced trapping of Pgp.

Parameter	Value	System	[reactant] (mM)				[Ver] (µM)	Ref
			ATP	ADP	P_i_	V_i_		
**for ATP**								
*k*_cat_	4.9 s^−1^	P					0	[8]
	9.2 s^−1^	P					50	[8]
*K_d_*	460 µM	P^a^^,^ b					0	[17]
	280 µM	P^b^					0	[20]
	870 µM	P^a^^,^ c					0	[29]
*K_m_*	800 µM	P					0	[8]
	800 µM	P					50	[8]
	1500 µM	PM			0		10	[14]
	1200 µM	PM			200		10	[14]
	1400 µM	PM					10	[5]
	330 µM	P					5	[9]
	300 µM	P					10	[9]
*IC*_50_ for trapping	9 µM	PM	1.0			0.2	10	[23]
% trapping	>90%	PM	1.0			0.2	10	[23]
*t*_½_ for trapping	∼10 s	PM	1.0			0.2	10	[23]
*t*_½_ for ATPase recovery	84 min^e^	PM	± 10.0					[23]
**for ADP**								
*K_d_*	330 µM	P^b^					0	[20]
*K_i_*	350 µM	PM					10	[5]
	700 µM	PM					50	[8]
*IC*_50_ for trapping	15 µM	PM				0.2	10	[23]
% trapping	>90%	PM		1.0		0.2	10	[23]
*t*_½_ for trapping	4.8 min	PM		1.0		0.2	10	[23]
*t*_½_ for ATPase recovery	84 min^e^	PM	± 10.0					[23]
**for P_i_**								
*IC*_50_ for hydrolysis	200 mM	PM	1.0				10	[14]
*K_i_* for trapping	100 mM	PM	0.2			0.2	10	[14]
	70 mM	PM		0.2		0.2	10	[14]
% trapping	85%	PM	1.0		10	0.2	10	[23]
	81%	PM	1.0		200	0.2	10	[23]
**for V_i_**								
*IC*_50_ for	12 µM	PM^f^	2.5				10	[5]
hydrolysis/trapping	4 µM	PM	1.0				10	[23]
	9 µM	P	0.2				50	[8]
	4 µM	PM	0.2		0		10	[23]
	8 µM	PM^d^	0.2		200		10	[23]
	9 µM	PM		0.2	0		10	[23]
	26 µM	PM^d^		0.2	200		10	[23]

Compilation of the values reported for some of the parameters that describe the ATPase and V_i_ trapping properties of Pgp from Chinese hamster ovary cells.

P, solubilized Pgp; PM, plasma membrane; Ver, verapamil. Unless otherwise stated, *T* = 37°C.

a MIANS-labeled Pgp;

b *T* = 22°C;

c *T* = 10°C;

d Corrected for ionic strength;

e Trapping with 200 µM V_i_ and nucleotide;

f 5 mM ATP + 2.5 mM Mg^2+^.

## Results

### Setting and Evaluation of the Elemental Catalytic Cycle

The steady-state solutions of the biochemical variables for the *Elemental Cycle* correspond to the following expressions
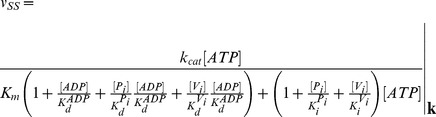
(1)

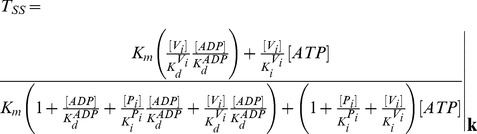
(2)with the steady-state concentration vector defined by 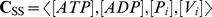. Eqs. 1–2 can be evaluated under the assumption that 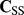 is almost identical to 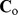 under the experimental conditions, e.g. using low [*E*]*_t_*, monitoring the initial rate of activity, and/or measuring the initial rate in the presence of an ATP-regenerating system. In both expressions, the thermodynamic parameters are defined by 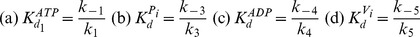
(3)and the kinetic parameters by
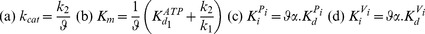
(4)where the factors *α* and *φ* are defined as
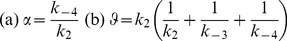
(5)


**Figure 3:**
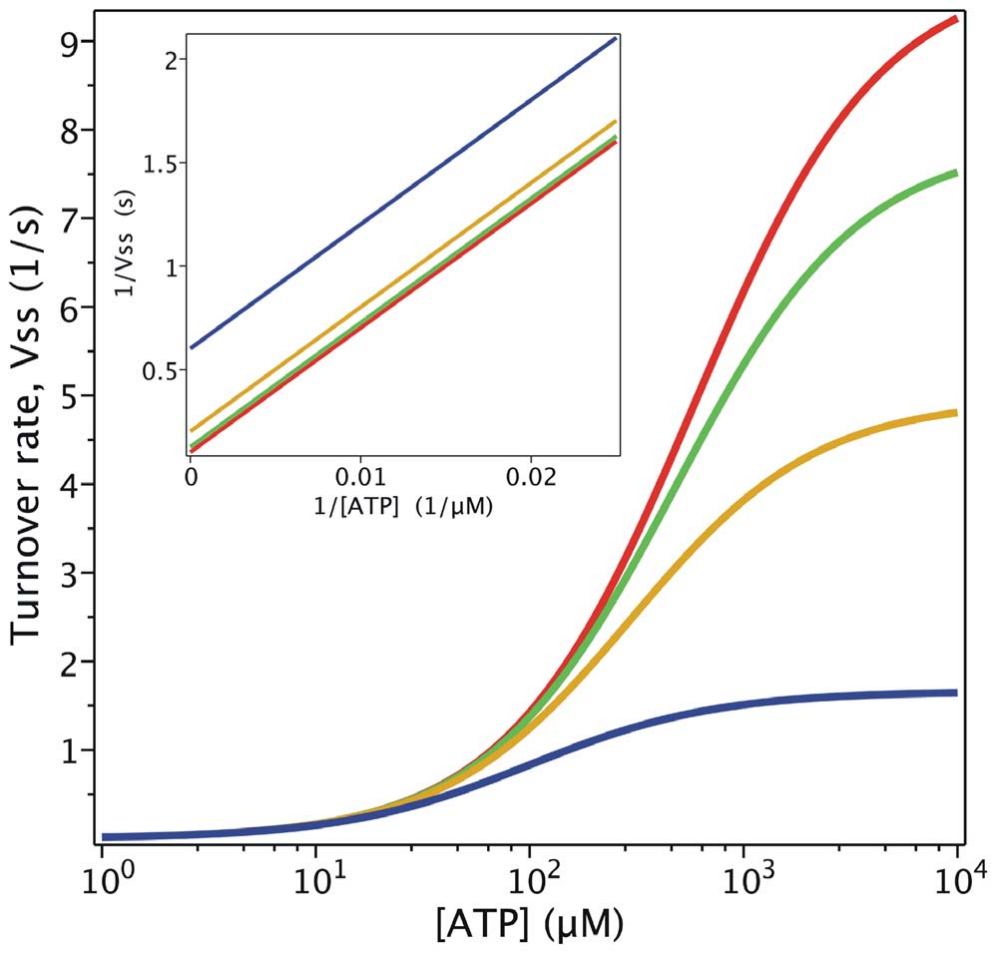
Effect of P_i_ on Pgp ATPase activity. Semi-log plot from the evaluation of 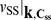 with 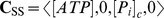 for 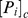 = 0 (red), 50 mM (green), 200 mM (yellow) and 1000 mM (blue). Inset: double-reciprocal plot with ATP concentrations ranging upwards from 100 µM. Values of **k** are given in **Table 2**.

From these basic parameters we derived the phenomenological parameters, 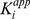 and *IC*_50_, for the observed steady-state turnover rate and trapped fraction. However, in the absence of rapid kinetic data for Pgp-mediated ATP hydrolysis, it was necessary to use an arbitrary setting for the vector **k.** Although, the exact values of the vector **k** are unknown, the settings specified here were designed to reproduce as closely as possible the published experimental data in **Table 1**. The rationalization of the *Elemental Cycle* rate constants is as follows:

- The setting of *φ* was a key element in the modelling. By definition, its value is >1, depending on the values of 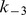 and 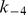 relative to 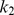 (Eq. 5b). Since P_i_ has low affinity (see (v)), 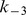 will be high (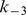
>>1 s^−1^), so the second term in Eq. 5b will be negligible. In addition, 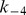
was set to the same value as 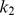 (see Discussion). From both considerations, *φ* takes the value of ∼2, however, its validation comes from the reciprocal constraints imposed by the interplay of the thermodynamic and kinetic parameters (Eq. 4).
- The observed catalytic rate, 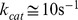 is equivalent to *v_max_* of ∼10 µmol P_i_ mg^−1^min^−1^ [8]. Since two ATP are hydrolyzed per Pgp in the full catalytic cycle (see *Alternating Cycle* below) the rate constant for the hydrolytic step was set to 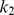 = 20 s^−1^ (Eq. 4a).
- In the absence of products and inhibitors, Eq. 1 presents the characteristic hyperbolic behavior observed for the ATP dependence of ATP hydrolysis by Pgp, according to
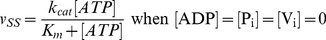
(6)with parameters within the range reported: a maximal turnover rate of 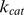, and a high consensus Michaelis-Menten constant of 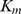
≅588 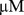 for verapamil-stimulated Pgp [5], [8]. The low affinity of ATP was set at 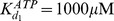, based on a reference value of ∼1 mM for the effect of ATP on inhibition of labeling of NBD1 by 8-azido-ATP [16]. From the selected 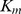 and 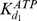
values, and *φ = *∼2, the ratio 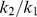 is predicted to be high (∼200, Eq. 4b), from which the rate constant for the association of ATP is estimated to be *k*_1_ = 0.1 µM^−1^s^−1^.
- ADP was reported to compete with ATP for the nucleotide-binding site [5], [9]. Effectively, the mathematical model predicts pure competitive inhibition behavior of ADP on ATP hydrolysis (**Figure S1**) according to
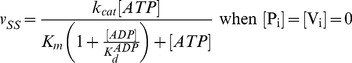
(7)with 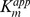 increasing with ADP concentration. Eq. 7 states that the ADP inhibition constant for ATP hydrolysis, 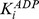, indeed corresponds to the ADP affinity, 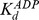. Thus, for a given 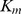, the ADP affinity is constrained by the observed 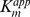. Herein, 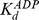
was set at 500 µM, which is compatible with the experimental value.
- Given 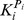 = 204 mM, which is close to the reported value [14], **Figure 3** shows the simulated output of the hydrolytic activity when [ATP] and [P_i_] were varied, according to
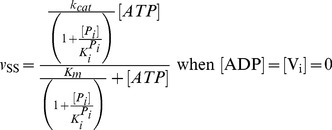
(8)

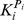
constrains the value of the affinity constant for binding of *P*_i_ to the complex 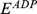. Thus, given the value of *φ* and *α* = 1 (i.e. 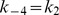), 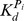
is estimated to be 100 mM (Eq. 4c).
- Inhibition of ATPase activity following reaction with V_i_ has been extensively studied. It was demonstrated early on that the trapped species is the long-lived Pgp≅ADP≅V_i_ complex, independent of the nucleotide used, and that the release of ADP correlates well with the slow reactivation of the enzyme [23]. The initial rate of ATPase activity, measured after rapid (∼30 s) removal of unbound ligands, is approximately proportional to the relative concentration of untrapped enzyme. From Eq. 1, the ATP dependence of V_i_ inhibition is described by
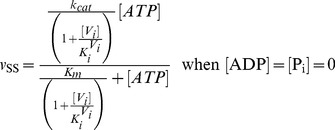
(9)

**Figure 4:**
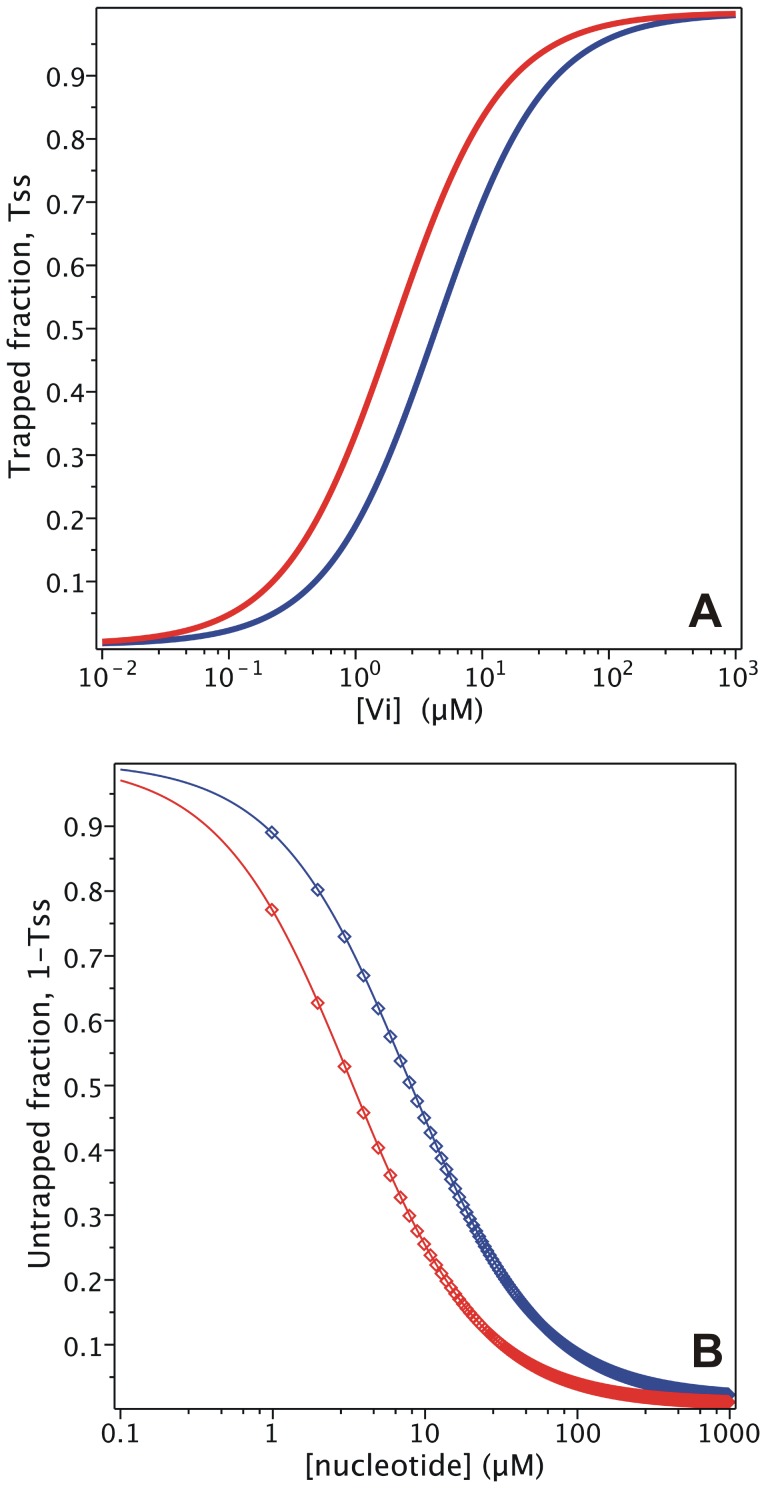
V_i_ interaction with nucleotides in the trapping of Pgp. (A) Semi-log plot of the V_i_ concentration dependence of the trapped enzyme fraction with 1000 µM ATP (blue symbols) or 1000 µM ADP (red symbols), from the evaluation of 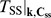 with 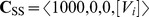 and 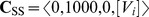, respectively. (B) Semi-log plot of the nucleotide dependence of the fraction of free enzyme, with 200 µM V_i_ and either ATP (blue symbols) or ADP (red symbols), evaluating 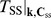 with 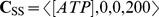 and 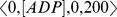, respectively. Lines are the best fits to the Hill equation with *n* = 1. Values of **k** are given in **Table 2**.

According to Eq. 9, 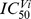 corresponds to the solution of the following equation for [*V_i_*]
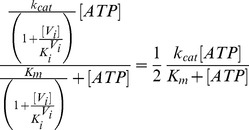
(10a)resulting in
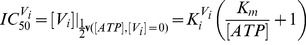
(10b)which is the same as 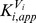 for the V_i_ concentration dependence of ATP hydrolysis. Setting 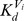 = 1.33 µM, yields 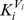 = 2.72 µM (Eq. 4d) and the 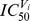 corresponds to 4.32 µM at 1 mM ATP (see **Figure 4A**). A value close to 4.0 µM was reported for half-maximal inhibition of Pgp ATPase activity by V_i_ under the same conditions [23]. Similarly, the *trapped fraction* variable was a query in our analysis. The steady-state concentration of trapped enzyme defined in Eq. 2 follows a hyperbolic curve as the ATP concentration increases, according to
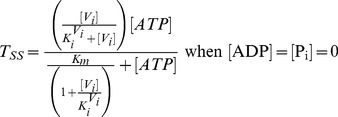
(11)


At saturating V_i_ concentration (e.g. 200 µM), the 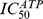 is indeed the “
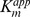
” term in Eq. 11, which for the given 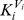 value yields 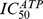 = 7.9 µM, close to the reported value of 9.0 µM [23] (see **Figure 4B** for the untrapped fraction).

- Given the value of 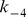, and 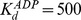 µM, 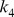 was estimated to be 0.04 µM^−1^s^−1^ (Eq. 3c). This does not agree with 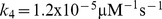 as suggested by Urbatsch et al. [23] based on the kinetics observed for ADP trapping following the route 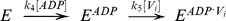. The latter 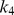 value would yield a forward rate constant 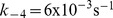 (given 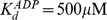) which is far too low to be compatible with the 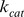 of 10 s^−1^ observed for ATP hydrolysis. Therefore, for our simulation, the reported 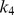 was ignored and we used instead the value imposed by the mutual interplay among all the parameters.
- It has been reported that V_i_-induced trapping is completed in about 10 s (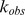
∼0.3–0.4 s^−1^) by the pathway 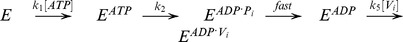 (**Figure 1**), with 200 µM ATP and V_i_ [23]. Thus, for the given 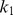 (and 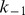) and 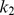, reaction with V_i_ becomes the rate-limiting step, with a calculated lower limit of about 0.0015 µM^−1^s^−1^. However, taking into account the other pathway for breakdown of the intermediate 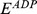 in the absence of *P*_i_ (
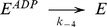
, with 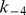 = 20 s^−1^), 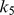 was here set to 0.015 µM^−1^s^−1^.

**Table 2** presents the assigned values of the rate constants (using the arguments above) for the 9 unidirectional reactions defining the vector **k**, and the derived dissociation constants, *K_d_*, for the 4 bidirectional steps (Eq. 3). Once defined, **k** was kept constant for the rest of the simulations and validations.

**Table 2:** Rate constants for the *Elemental Catalytic Cycle*.

Process	Parameter	*K*_d_	*k*
ATP association	*k*_1_		0.1 µM^−1^s^−1^
ATP dissociation	*k*_−1_		100 s^−1^
	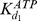	1000 µM	
ATP hydrolysis	*k*_2_		20 s^−1^
P_i_ association	*k*_3_		5 mM^−1^s^−1^
P_i_ dissociation	*k*_−3_		500 s^−1^
	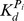	100 mM	
ADP association	*k*_4_		0.04 µM^−1^s^−1^
ADP dissociation	*k*_−4_		20 s^−1^
	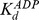	500 µM	
V_i_ association	*k*_5_		0.015 µM^−1^s^−1^
V_i_ dissociation	*k*_−5_		0.020 s^−1^
	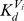	1.3 µM	

Rate constants defining the vector **k** for the reaction scheme shown in **Figure 1**. The nomenclature of the subscripts is follows: (±1) for the ATP equilibrium, (+2) for the hydrolytic step, (±3) for the P_i_ equilibrium, and (±4) for the ADP equilibrium. A positive sign is used for association reactions, a negative sign for dissociation reactions.

The effect of P_i_ on ATPase activity was a key element in the validation of the model. It was previously reported that P_i_ behaves as a mixed-type inhibitor of ATP hydrolysis [23], where 200 mM P_i_ reduces the apparent *v*_max_ by 50%, while the apparent *K_m_* is reduced by just 20% [14]. In contrast, according to Eq. 8, 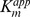/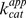 (the slope of the lines in the Lineweaver-Burk plot, see **Figure 3** inset) is independent of inhibitor concentration, since 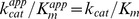, so that P_i_ behaves instead as an uncompetitive inhibitor, which is incongruent with the reported data.

Another inconsistency between the output of the model and experimental data comes from trapping with ADP. It has been reported that 15 µM ADP produces half-maximal inhibition in the presence of 200 µM V_i_ [23]. According to Eq. 2, the ADP dependence of the trapping is defined by
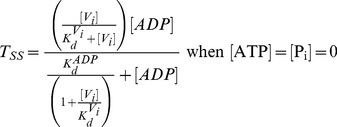
(12)where at saturating V_i_, the “
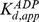
” term corresponds to 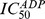 (as with ATP dependence, see Eq. 11). Thus at 200 mM V_i_, given the 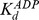 and 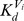 values, the calculated 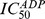 is 3.31 µM, which is 5-fold lower than the reported value [23].

**Figure 4B** presents the simulated nucleotide dependence of the untrapped (free) fraction, *1-T_SS_*, for both cases given by the model in **Figure 1**. Fitting of the synthetic data gave a Hill number of *n* = 1 for both ATP and ADP, which is expected for binding of just one nucleotide according to Eqs. 11 and 12. However, the behavior reported experimentally was a steeper concentration dependence for both ATP and ADP [23].

**Figure 5:**
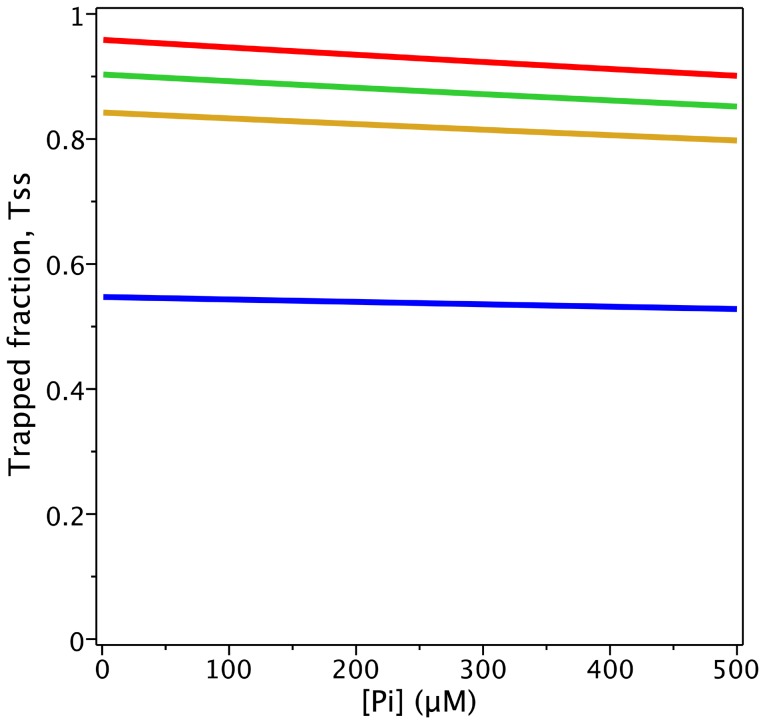
Protection of Pgp from V_i_ trapping by P_i_. Plot of the P_i_ concentration dependence of the trapped enzyme fraction with 100 µM V_i_ and different ATP concentrations, from the evaluation of 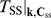 with 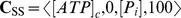 for [*ATP*]*_c_* = 1000 µM (red), 200 (green), 100 (yellow) and 20 µM (blue). Values of **k** are given in **Table 2**.

The ability of P_i_ to protect Pgp from V_i_ trapping was also tested using the model. It was reported that in the presence of 200 µM V_i_, protection by 200 mM P_i_ is negligible at 1 mM ATP, but becomes significant at lower ATP concentrations [23]. This differential P_i_ protection effect depending on ATP concentration could not be reproduced by the model in **Figure 1**. The evaluation of *T_SS_* as a function of [ATP] and [P_i_], in the presence of 100 µM V_i_, is plotted in **Figure 5**, which shows lines of similar slope, and ATP dependence opposite to that observed experimentally, i.e. the slopes decrease at lower ATP concentration. From Eq. 2, 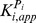 decreases with increasing [ATP] according to
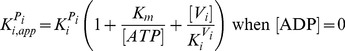
(13)


**Figure 6:**
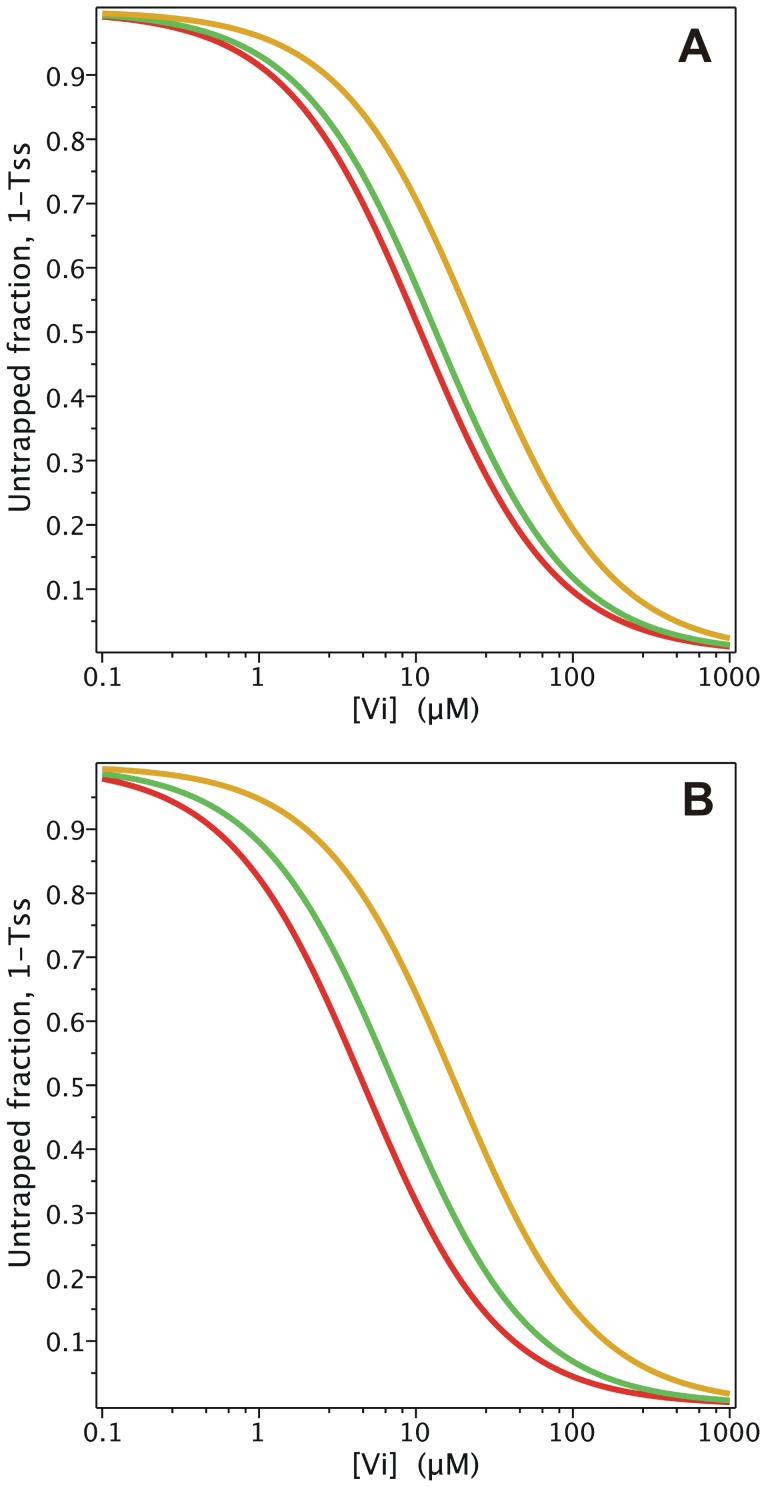
Effect of P_i_ on the V_i_ dependence of trapping. Semi-log plot of the V_i_ concentration dependence of the untrapped enzyme fraction incubated with (A) 1000 µM ATP or (B) 1000 µM ADP, from the evaluation of 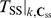 with 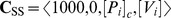 and 
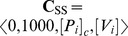
, respectively, for [*P_i_*]*_c_* = 0 (red), 200 µM (green), and 1000 µM (yellow). Values of **k** are given in **Table 2**.

Another discrepancy between the behavior of the model and experimental data comes from the interaction of V_i_ and P_i_ with the 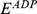
complex. From the evaluation of *T_SS_* at 200 µM ATP (**Figure 6A**) or ADP (**Figure 6B**) as a function of [*V_i_*] and [*P_i_*], the competitive interaction reported for these two anions is evident, according to
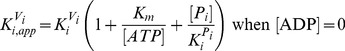
(14a)

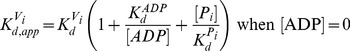
(14b)


However, fitting of the synthetic data in **Figure 6** to an expression using an effective inhibition constant for P_i_, 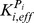, according to
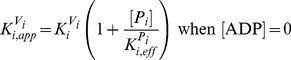
(15a)

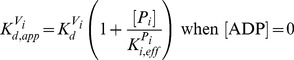
(15b)yields values of 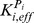 = 51.8 and 45.4 mM, for trapping with 200 µM of ATP and ADP, respectively, half the reported values of 100 and 70 mM, respectively, after correction for ionic strength [14]. The experimental values might be matched by increasing 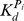, but then the capacity of P_i_ to inhibit hydrolytic activity would be affected (see (*v*)).

**Figure 7:**
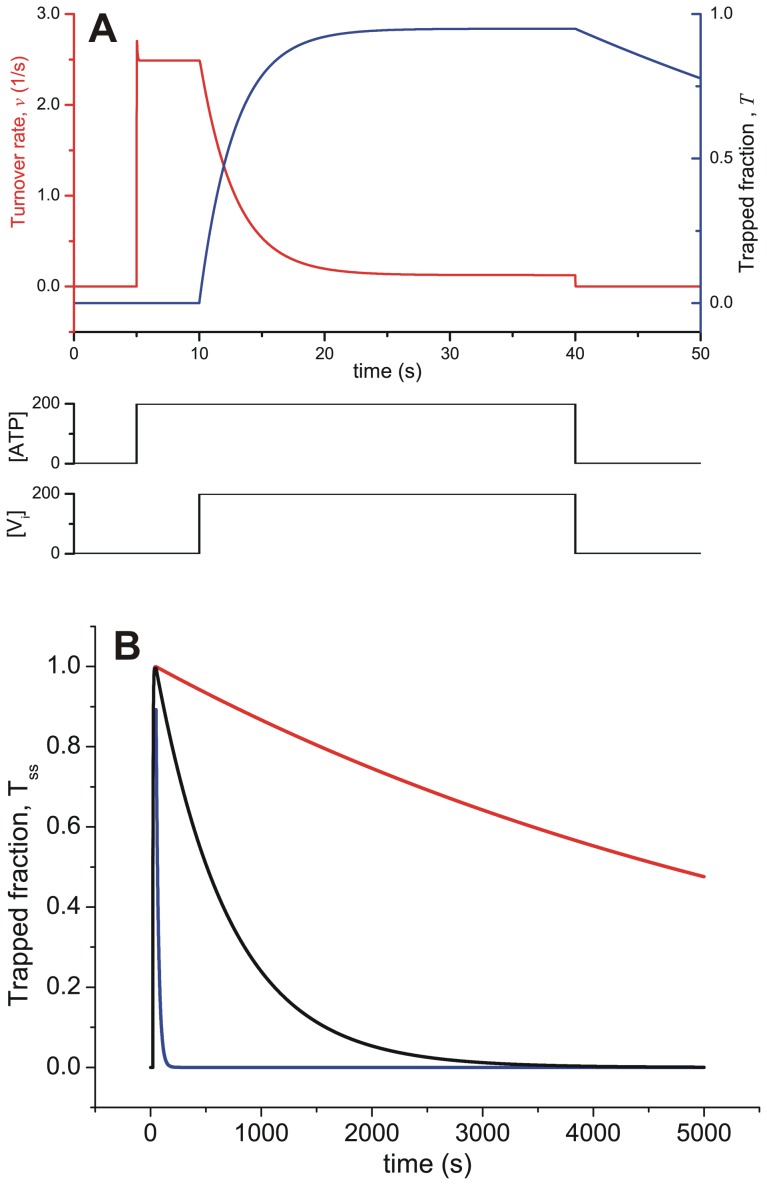
Time-course of ATPase activity and formation of trapped Pgp. (A) Transient behavior of ATPase activity (red) and the fraction of trapped enzyme (blue), evaluating 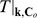 with 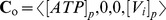 at the indicated concentration pulses of ATP and V_i_. (B) Time-course of the fraction of trapped Pgp according to V_i_ affinity. Transient behavior of the fraction of trapped enzyme on incubation with ATP and V_i_, evaluating 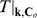 with 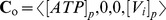 for pulses of 200 µM ATP and V_i_ of 50 s duration (not shown). Each curve corresponds to 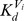  =  3 µM (blue), 0.1 µM (black) and 0.01 µM (red). Values of **k** are given in **Table 2**; [*P*]*_t_* = 0.25 µM.

Considering the time domain, **Figure 7A** shows the time-course of the overall activity and formation of the trapped species, for a pulse of ATP and V_i_. Thus, evaluating ***T*** with 200 µM [*ATP*]*_o_* and [*V_i_*]*_o_* (keeping both constant), the numerical simulation mimics the rapid formation of the trapped species (within 10 s) and the high steady-state fraction trapped that was reported in the literature [23]. However, the output of the model clearly disagrees with the reported transient kinetics of dissociation of the V_i_-trapped state. Experimentally, upon removal of unbound ligands, the observed slow dissociation has 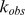 = 1.4×10^−4^ s^−1^ (τ_½_ = 87 min) [23], which correlates with the recovery of ATPase activity by the pathway 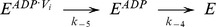 (**Figure 1**). The step 
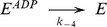
occurs at the rate of 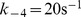, which is compatible with the turnover rate, and rules out this reaction as the rate-limiting step for recovery of activity. On the other hand, the step 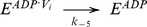 has a rate constant 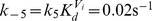, which is 140-fold higher than the observed dissociation rate. Thus, in order to match the observed kinetics of ATPase recovery either (i) the dissociation constant 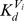 must be much lower than 0.01 µM (see **Figure 7B**), a value which is incompatible with the observed 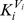 for trapping with ADP and ATP (see above), or (ii) the association constant 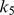 must be much lower than 0.015 s^−1^, which is incompatible with the fast formation of the trapped species (**Figure 7A**).

The slow recovery of ATPase activity from the trapped species might be explained by the existence of several hidden transitions in the overall reaction 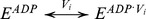. This possibility was tested by adding a step with a low dissociation rate constant (<0.001 s^−1^) to explain the slow backward reaction to form 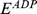. Effectively, the pathway for the trapping reaction was substituted by 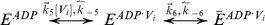, which describes consecutive equilibria with 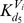 and 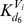
dissociation constants, respectively. In order to include a slow backward step and shift the equilibrium toward the species on the right, the new forward rate constant 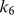 was set to 1×10^−3^ s^−1^ and the backward rate constant 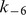 to 1×10^−4^ s^−1^ (yielding 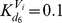), with a concordant increase of the V_i_ association equilibrium constant 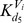 to ∼10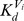. In this way, it would be possible to explain the slow recovery of ATPase activity, while the change in overall affinity of V_i_, 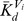, would not significantly affect the 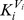 for trapping with ADP and ATP. However, inclusion of this additional step could still not explain the slow inhibition observed with ADP, by the pathway 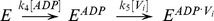. This issue will be considered further below.

### Construction of the Alternating Catalytic Cycles

In this section, we evaluate the *Alternating Catalytic Cycle* proposed by Senior et al. [25]. In our adaptation of the model (shaded cycle, **Figure 2**), the two equivalent forms of the enzyme, *E* and *F*, correspond to states of the enzyme with similar energetic and/or conformational states that differ only in the hydrolytic properties of their individual NBDs. This notation is necessary to distinguish between the two-nucleotide species, 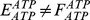, according to their NBD hydrolytic activity, i.e. the *E*-form is capable of hydrolyzing only the ATP molecule bound at NBD1 (but not at NBD2), and *vice versa* for the *F*-form, thus moving the enzyme symmetrically between both states. Two different models can account for the *E*/*F* forms; in both it is necessary to include ATP binding at each NBD of the bare enzyme as a first step (priming reaction) to get the initial intermediates of the cycle: (i) starting from the same conformer of the enzyme, *P*, recruitment of the NBDs to the nucleotide-bound state occurs randomly, with the probability of occupancy given by the intrinsic affinities of each NBD, so that 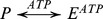
(where binding takes place at NBD1) or 
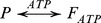
 (where binding take place at NBD2), or (ii) both conformers of the empty protein (*E* and *F*) co-exist, each exhibiting its own constitutive binding properties (*E* allows binding at NBD1, while *F* allows binding at NBD2); they may or may not be kinetically connected by the equilibrium 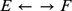. The kinetics of ATP hydrolysis and V_i_ trapping are identical in both models. For the sake of simplicity, we decided to work with the first model, with the conservation of mass given by
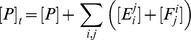



with *i,j* for: *none*, *ATP*, *ADP*, *ADP⋅P_i_* and *ADP⋅V_i_*, according to **Figure 2**. In this model, the intermediates 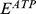 and 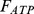 exhibit the same properties regardless of their origin, whether from the priming reaction or a later hydrolytic event. In this particular implementation of the *Alternating Cycle*, transformation between the two forms was achieved by exchange of ADP from/to the two-nucleotide intermediates to/from the one-nucleotide intermediates: 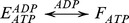 and 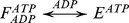, respectively. However, it would be equivalent to assign the transformation to either the hydrolytic step (which looks reasonable) or the P_i_ dissociation step, since in either case, the kinetic behavior of the system is the same. To maintain symmetry, this step was assigned to the dissociation/association of ADP, which is the last hydrolysis product to leave the NBD. In addition, it was necessary to include the trapping reactions with V_i_ for each half-cycle (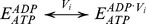 and 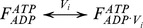).

**Figure 8:**
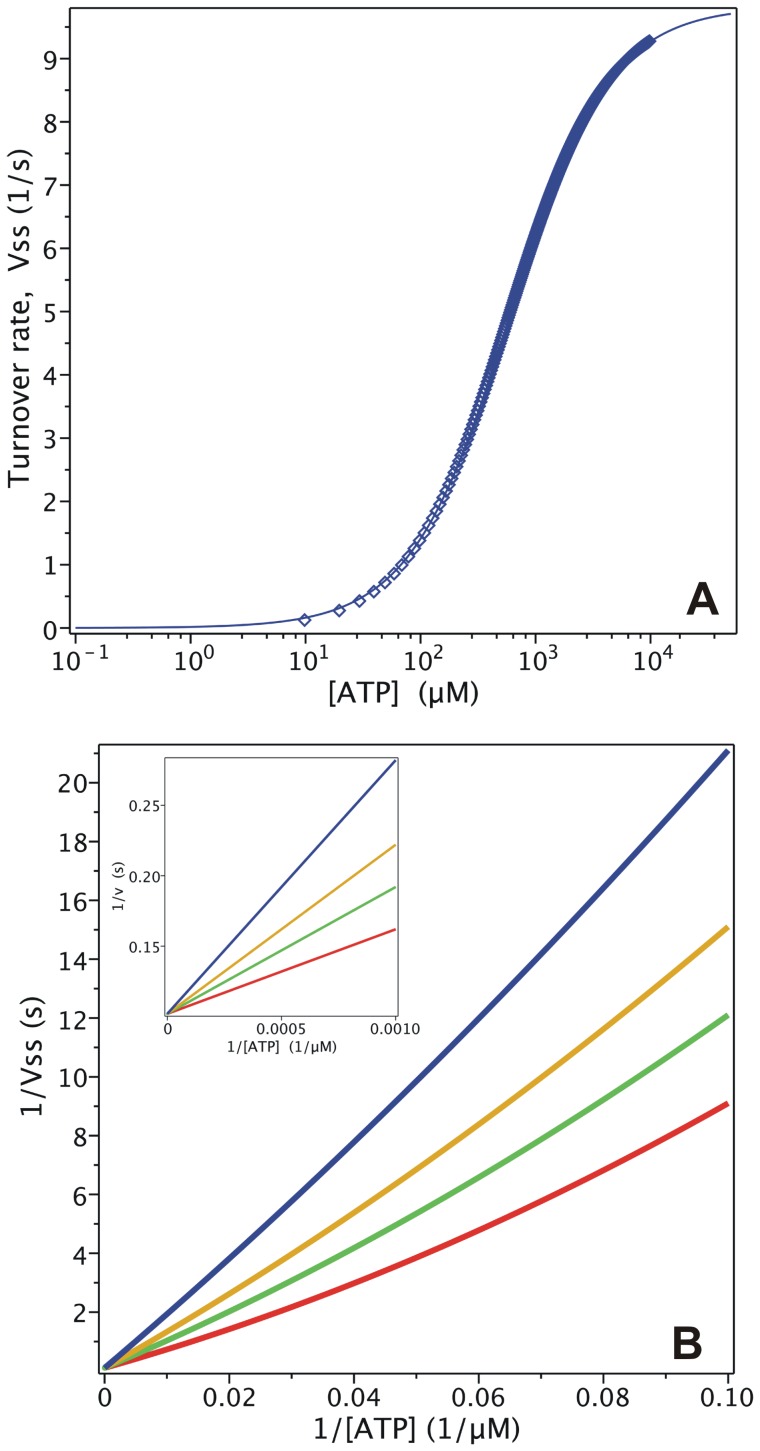
Steady-state simulation of the *PE Alternating Cycle*. (A) ATPase activity. Semi-log plot of ATP turnover rate (symbols) from the evaluation of 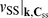 with 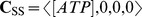. The line is the best fit to a hyperbolic equation. (B) Inhibition by ADP. Double-reciprocal plots for ATP turnover rate from the evaluation of 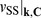 with 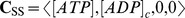 for [*ADP*]*_c_* = 0 (red), 250 µM (green), 500 µM (yellow) and 1000 µM (blue), with ATP concentration up to 100 µM. Inset: Double-reciprocal plots with ATP concentrations ranging upwards from 100 µM. Values of **k** are given in **Tables 2** and **3**.

It is remarkable that neither of these two obvious steps (the priming and trapping reactions) has been depicted explicitly in any reaction scheme that considers both half-cycles simultaneously. The former was added later for first time by Urbatsch et al. [32], who considered that both NBDs binds ATP independently (priming reaction) and then come together (dimerization) to form the species with two bound ATP (although their concept was different from the one proposed here, see Discussion). We describe this new kinetic model, with both priming and trapping reactions (grey cycle plus blue reactions in **Figure 2**), as the *Partial-Extended (PE) Alternating (Catalytic) Cycle*. Any differences between the properties of the *PE Alternating Cycle* and a tandem repeat of the *Elemental Cycle*, can arise only from these additional reactions steps. Therefore, we were interested in evaluating the influence of the priming reactions in the ATP dependence of several observables. The steady-state solutions of the biochemical variables for the *PE Alternating Cycle* correspond to the following expressions 
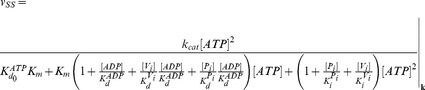
(16)

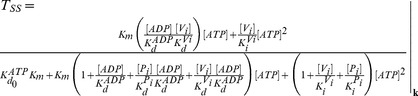
(17)with the steady-state concentration vector defined by 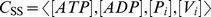. For the evaluation of Eqs. 16 and 17, it was assumed that 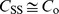, as explained earlier. For this model, the velocity and trapping equations are no longer hyperbolic. For example, the ATP dependence of the turnover rate now follows a quadratic equation given by
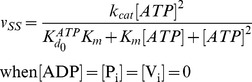
(18)where 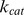 and *K_m_* are the parameters corresponding to the previous model (the *Elemental Cycle*), and the ATP affinity of the bare enzyme is defined by 
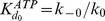
. It is important to note that, whatever the relative value of 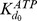, Eq. 18 can be adequately fitted to the function
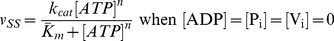
(19)with *n*≥1 and 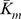 being an effective Michaelis-Menten constant. It is interesting to note that if 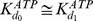, the deviation from hyperbolic is appreciable only at high ATP concentrations. On the other hand, if we consider a much lower 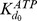 (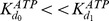), *n* approaches 1 and the deviation from hyperbolic is negligible, and only observed at very low ATP concentrations. Because the majority of reports describe Pgp ATPase activity as Michaelian, we set the value of 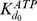 in the µM range. This value also matched the low *K_d_* value for the poorly-hydrolysable analog ATPγS [29] and other experimental evidence [33] explained by the model (see Discussion). Thus, simulating the *PE Alternating Cycle* with the parameters in **Tables 2** and **3**, the fitting that describes the ATP dependence of activity is an effective single 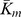 of 596 µM for *n* = 1, a value very close to that obtained for the *Elemental Cycle* (**Figure 8A**).

**Table 3:** Rate constants for the priming reaction of the *PE Alternating Cycle*.

Process	Parameter	*K*_d_	*k*
ATP association	*k*_0_		10 µM^−1^s^−1^
ATP dissociation	*k*_−0_		50 s^−1^
	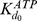	5 µM	

Rate constants defining the vector **k** in conjunction with the rates in **Table 2**, for the blue reactions in **Figure 2**. The nomenclature of the subscripts is as follows: (±0) for the ATP priming equilibrium.

**Figure 9:**
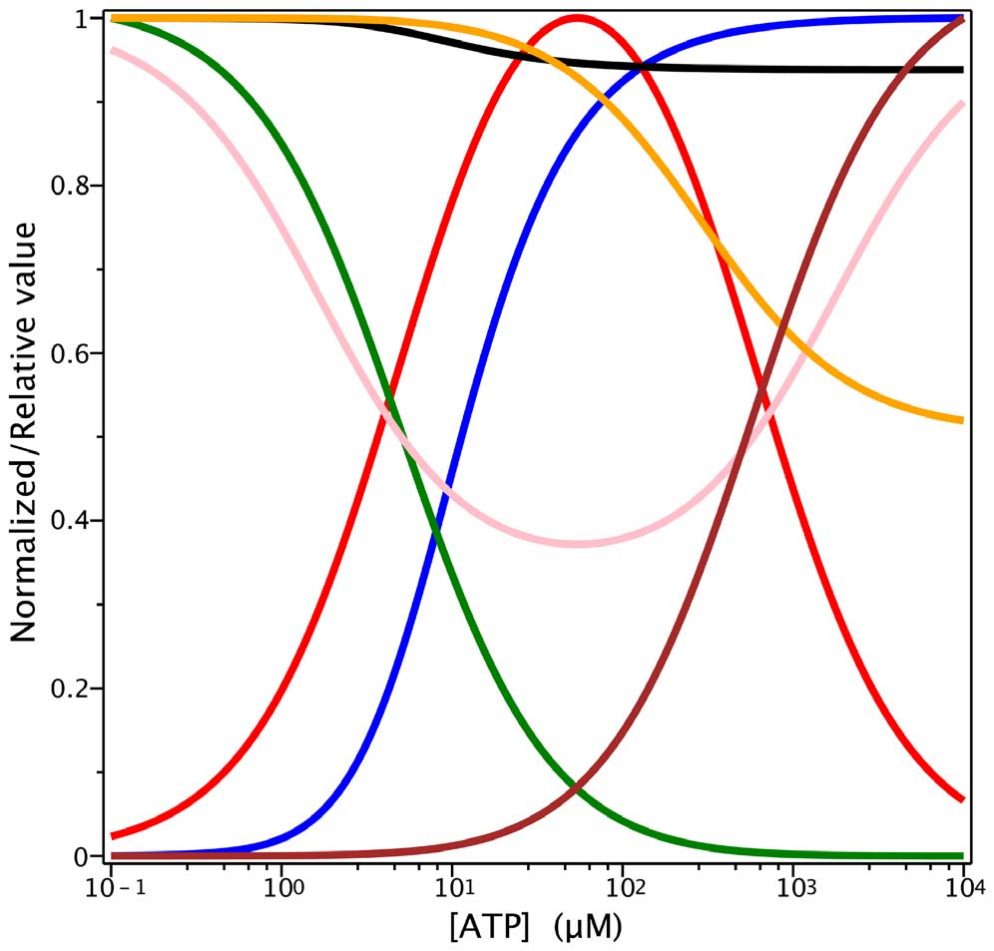
ATP dependence of several variables according to the *PE Alternating Cycle*. Semi-log plots of the steady-state ATP dependence of the normalized concentrations of (i) one-nucleotide species (red): 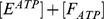 with 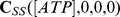 (ii) two-nucleotide species (brown): 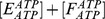 with 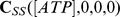 (iii) bare enzyme (green): 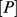 with 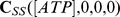 and the relative hydrolytic activity, by evaluating 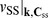
for (*iv*) ADP inhibition (pink): 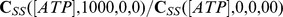 (v) P_i_ inhibition (yellow): 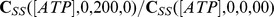 and the normalized trapped fraction, by evaluating 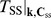
for (vi) trapped species (blue): 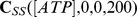 (vii) P_i_ protection of V_i_-trapping (black): 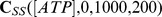 Concentration values for 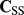 are given in µM except for P_i_, which are in mM. Values of **k** are given in **Tables 2** and **3**. [*P*]*_t_* = 0.5 µM.

The interaction with ADP is now no longer one of simple competition (**Figure 8B**), and is described by
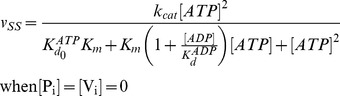
(20)unless we include a pathway for the reaction 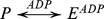 and its equivalent for the *F*-form (shown in **Figure 2** in red, but not considered at this stage). Nevertheless, at high ATP concentration (e.g. [ATP] >100 µM >>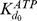), the behavior is apparently competitive, as the literature indicates (**Figure 8B** **, inset**), since the interaction occurs mainly *inside* the catalytic cycle (where both nucleotides compete for the vacant site in 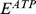
and 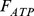), and the concentration of the bare enzyme, *P*, is negligible at that ATP concentration (see **Figure 9**).

As expected, the observed properties with respect to P_i_ remained constant, with a effective inhibition constant, 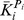 of ∼200 mM, since the relationship between the phosphate binding step and the hydrolytic step is conserved between the *Elemental Cycle* (or tandem repeats of it) and the *PE Alternating Cycle*. However, the double-reciprocal plot of the ATP dependence of activity (not shown) has an upward curvature given by
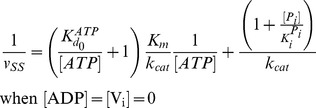
(21)


revealing that indeed the slopes are P_i_-independent (as for the *Elemental Cycle*), but are now affected by the ATP concentration.

**Figure 10:**
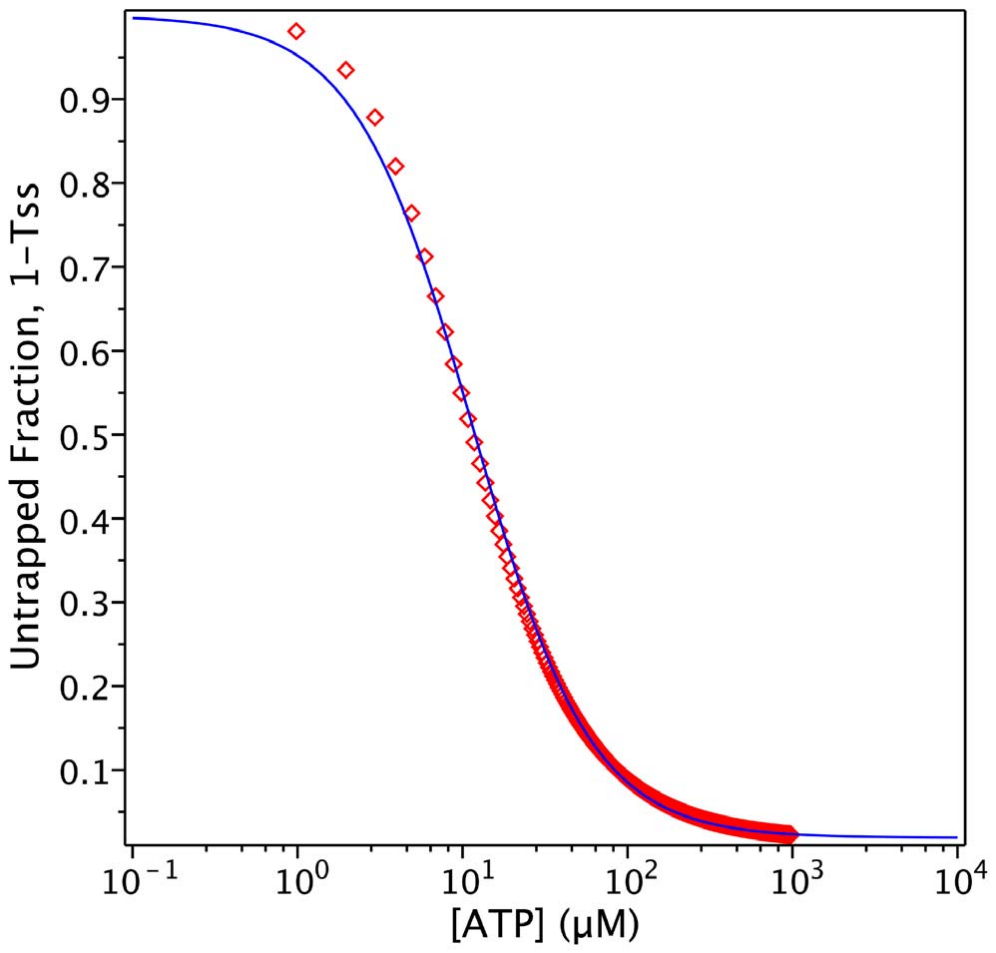
Steady-state simulation of the *PE Alternating Cycle*. ATP dependence of trapping. Semi-log plot of the ATP concentration dependence of the untrapped enzyme fraction (red symbols) on incubation with 200 µM V_i_, from the evaluation of 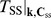
with 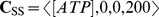. Blue line is the best fit to the Hill equation, with *n* = 1.21. Values of **k** are given in **Tables 2** and **3**.

V_i_ also behaves similarly in the *PE Alternating Cycle*, inhibiting ATPase activity at low concentrations. As indicated previously, the *Alternating Cycle* by itself cannot explain the cooperativity found in the nucleotide dependence of V_i_ trapping. This cooperative behaviour arises because of the priming reaction in the *PE Alternating Cycle*. From Eq. 17, producing synthetic data for the untrapped fraction, 1-*T_SS_*, with parameter values of 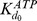 = 5 µM and 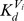 = 1.33 µM, (**Figure 10**), and performing an unweighted fitting according to
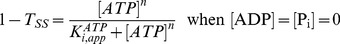
(22)we obtained a Hill number of *n* = 1.21 and 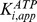 or 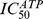 of ∼20 µM. Unfortunately, there is no experimental data published for hamster Pgp to compare with the Hill number obtained by simulation. For reference, 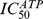 for the closely-related mouse Pgp was reported to be 18 µM, with *n* = 1.7 [32]. Since Eqs. 16 and 17 describe with more accuracy the turnover rate and trapped fraction, they should be used to set the value of 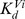, rather than Eqs. 1 and 2, as was done in the previous section.

For the priming binding reaction with ATP, 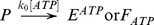, the association rate constant was set to 100-fold the value for the corresponding rate *inside* the cycle, 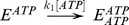 (and the *F*-form equivalent), which is 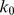 = 10 µM^−1^s^−1^. Thus, the priming reaction would not limit the establishment of steady-state catalysis. In addition, this relatively high value for the priming association rate constant permits further decreases to allow our model to explain the observed impairment in trapping behavior in some systems [29], [34].

However, some important experimental data still remain unexplained according to the *PE Alternating Cycle*: (i) the slow kinetics of V_i_ inhibition with ADP, (ii) the slow kinetics of reactivation of ATPase activity, and (iii) the stoichiometry of 1∶1 Pgp:nucleotide in the trapped species, where ADP is trapped with V_i_. Indeed, according to the kinetic reactions in the *PE Alternating Cycle* (**Figure 2**, grey cycle plus blue reactions only), the trapped species should contain both ATP and ADP (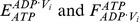), since there is no direct pathway to release ATP before V_i_. Furthermore, according to this scheme, the bound ATP would be hydrolyzed when the enzyme re-enters the cycle upon V_i_ release.

As pointed out above, there is the need to add plausible steps that account for the observed kinetics of trapping and release of both nucleotides. If we now incorporate the red reactions, **Figure 2** outlines a minimal reaction pathway, including (i) adaptation of the basic alternating cycle proposed by Senior et al. [25] (grey cycle and trapping reaction with V_i_), (ii) the priming reaction with ATP (blue reactions), and (iii) the priming reaction with ADP and a pathway for the release of ATP from the two-nucleotide trapped species (red reactions), by an independent pathway different from the catalytic reactions. The model for the *Extended Alternating (Catalytic) Cycle* was simulated only by numerical methods, using the rate constants in **Tables 2** **,** **3** and **4**.

**Table 4:** Complementary rate constants for the *Extended Alternating Cycle*.

Process	Parameter	*K*_d_	*k*
ADP association	*k*_4a_		0.04 µM^−1^s^−1^
ADP dissociation	*k*_−4a_		2 s^−1^
	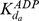	50 µM	
V_i_ association	*k*_5a_		2×10^−5^ µM^−1^s^−1^
V_i_ dissociation	*k*_−5a_		1×10^−4^ s^−1^
	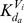	5 µM	
ATP association	*k*_1a_		1×10^−5^ µM^−1^s^−1^
ATP dissociation	*k*_−1a_		3 s^−1^
	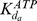	30 mM	

Rate constants defining the vector **k** in conjunction with the rates in **Table 2** and **Table 3**, for the red reactions in **Figure 2**. The nomenclature of the subscripts is the same as in **Table 2**, with the addition of the suffix *a* to identify this pathway.

**Figure 11:**
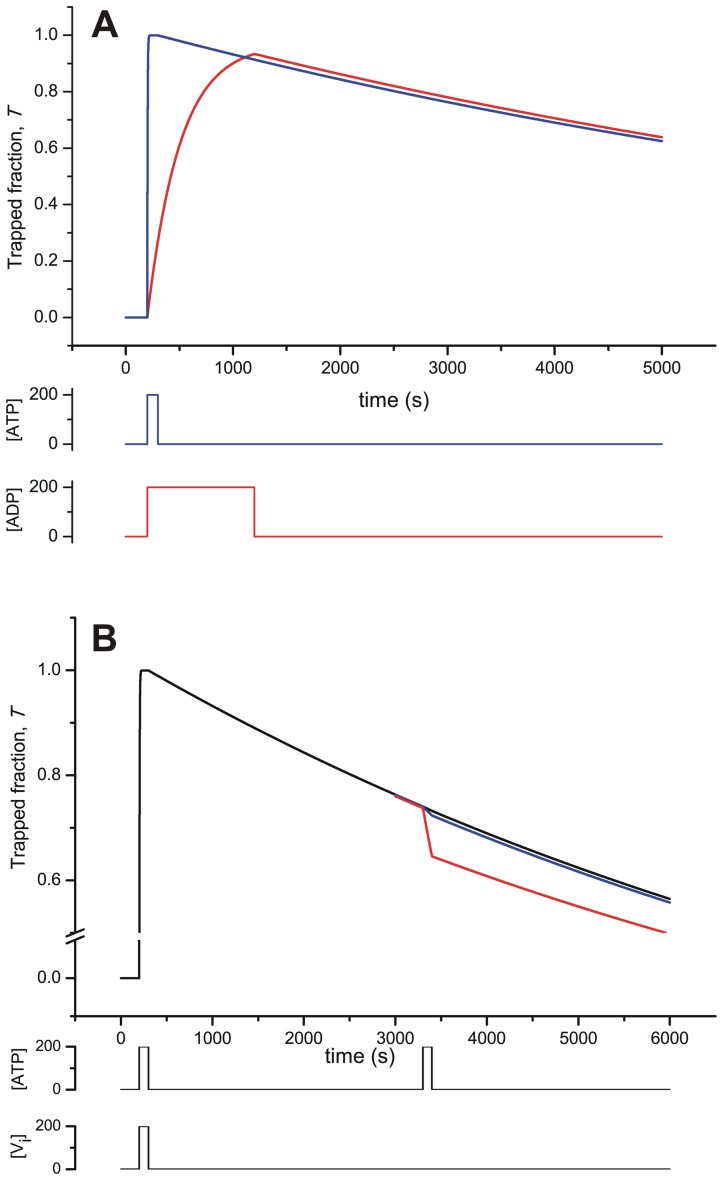
Time-course simulation of the *Extended Alternating Cycle*. (A) Time-course of V_i_ trapping. Transient behavior of the trapped fraction evaluating 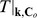 with 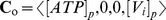 and 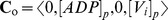 at the indicated concentration pulses of V_i_ and ATP (100 s; blue) or V_i_ and ADP (1000 s; red), respectively. (**B**) Time-course of decay of the trapped species in the presence of ATP. Transient behavior evaluating 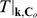 with 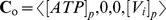 at the indicated concentration pulse of V_i_ and ATP (100 s), and a second pulse of ATP (100 s) during the recovery phase, by setting *k*_1a_ = 10^−3^ (red), 10^−4^ (blue), and 10^−5^ µM^−1^s^−1^ (black). The remaining values of **k** are given in **Tables 2**, **3** and **4**. [*P*]*_t_* = 0.5 µM.

**Figure 11A** presents the time-course of the concentration of total trapped species during exposure to V_i_ with ATP or ADP. Here, there is a noticeable difference in the rate of accumulation of the trapped species (the rising phase) depending on the nucleotide used, which can account for the different observed rates of catalytic inhibition for the two nucleotides [23]. In the presence of V_i_ and ATP, by the pathway 
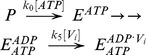
(and the *F*-form equivalent), the rapid trapping observed sets a relatively high value for *k*_5_. However, the fraction of trapped enzyme is now distributed between the one- and two-nucleotide species according to the equilibrium 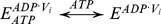
(and the *F*-form equivalent) defined by 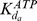 (see below). On the other hand, for V_i_ trapping in the presence of ADP, by the path 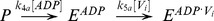 (and the *F*-form equivalent), the slower observed rate sets a lower value for the V_i_ association rate, *k*_5a_. The assignment of this latter step as being rate-limiting comes from the need to keep 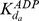 in the high µM range, so that 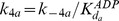 should not be low.

It has been reported that regardless of the nucleotide used, the trapped fraction corresponds to the ATP-free species, 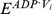 and 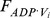 [23], therefore the dissociation constant 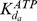
was set to a high value (high mM range). With both ATP and ADP trapping, after removal of the ligands (equivalent to turning off the nucleotide and V_i_ pulses in our simulation), the model exhibits characteristic reactivation of ATPase activity. This is inversely proportional to the trapped fraction of enzyme (the decay phase), and takes place at the same rate regardless of the nucleotide used, by the common path 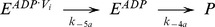 (and the *F*-form equivalent). This requires that when trapping with ATP, the small fraction of two-nucleotide species,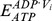 and 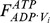, which can decay by two possible routes 
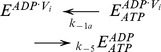
 (and the *F*-form equivalent), must drain mainly by the left-hand pathway; thus *k*_−1a_ was set to a higher value than *k*_−5_. With this setting, by making V_i_ dissociation rate-limiting, slow ligand release from the common species 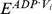 at a time constant of ∼1/*k*_−5a_ correlates well with ATPase recovery for trapping with both ATP and ADP.

In addition, it was interesting to investigate the effect of an additional pulse of ATP during the recovery phase. According to the model, in the absence of V_i_ the main trapped species is depleted by two possible routes: 
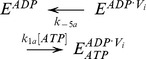
 (and the equivalent for the *F*-form). Since recovery of catalytic activity has the same slow kinetics whether or not ATP is present [23], then *k*_1a_<*k*_−5a_ (**Figure 11B**). This low rate constant for ATP association, *k*_1a_, agrees with the high 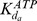
(for the given *k*_−1a_, in turn constrained as mentioned above). All this assumes that binding of ATP is the rate-limiting step in the forward recovery pathway, 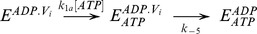, which is in concordance with the relatively high setting of *k*_−5_, constrained by the observed fast trapping and a µM value for 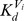, since 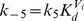.

## Discussion

Understanding the catalytic cycle of Pgp is essential to elucidate its transport mechanism. In spite of the efforts of several research groups over many years in providing good quality experimental data, no detailed kinetic analysis has yet been carried out. Consequently, some puzzling features of the system still remain unexplained, including: cooperativity of ATP hydrolysis at low ATP concentrations; mixed inhibition of ATPase activity by P_i_; the steep concentration dependence observed for V_i_ trapping with ADP/ATP; the kinetics of V_i_ release from the trapped species; the kinetics of V_i_ trapping with ADP; the relative *IC*_50_ values for V_i_ trapping using ATP/ADP; protection from V_i_-trapping by P_i;_ and the detection of one-nucleotide trapped species. In this work, we present a quantitative evaluation of the currently accepted models for ATP hydrolysis and V_i_ trapping, and assess their ability to explain the accumulated biochemical data. Using analytical and numerical methods, we evaluated the steady-state and the temporal behavior of the two main observable variables, the rate of ATP hydrolysis and the concentration of trapped enzyme. Thus, the basic reaction scheme for hydrolysis proposed by Urbatsch et al. [23], and its implementation in the *Alternating Catalytic Cycle* [25], were tested for their ability to reproduce the kinetic behavior of these variables.

The success and applicability of this mode of analysis depends critically on the set of kinetic parameters (rate constants) employed. Since such kinetic data does not currently exist, we established a coherent collection of rate constants that simultaneously matched both steady-state and temporal courses of all phenomenological and known thermodynamic properties describing catalysis and V_i_ trapping. This self-consistent set of parameters was obtained using the reciprocal constraints that impose: (i) the parameters that describe ATPase activity, i.e. 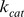, 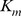 and Hill number *n*; (ii) reference values of 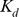 for nucleotides and P_i_; (iii) the kinetics and phenomenological *K_i_*/*IC*_50_ of products (ADP and P_i_) and inhibitors (V_i_) for hydrolysis and/or trapping; and (iv) the temporal course of V_i_ trapping and post-trapping recovery of ATPase activity (which is invaluable). It should be noted that some of these parameters are species-dependent. For example, 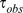 for trapping with V_i_ using ADP for mouse Pgp (ABCB1b/Mdr3) is an order of magnitude slower than that for hamster Pgp [32]. In this regard, **Table 1** compiles most of the parameters and observables reported for hamster Pgp (ABCB1a/Mdr1).

### The Steady-State Properties of the Elemental Cycle

As shown in Results, the output of this model is in agreement with the basic properties exhibited by an isolated half-cycle of ATP hydrolysis with respect to ATP dependence and competition by ADP. Our set of rate constants reported: (i) a high Michaelis constant (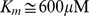) which, in combination with the relatively slow catalytic rate (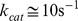), results in a low effective bimolecular rate constant 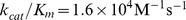; (ii) inhibition of ATPase activity by ADP at sub-mM levels (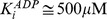); (iii) inhibition of ATPase activity by P_i_ at high mM levels (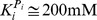); (iv) inhibition of ATPase activity by V_i_ at µM levels (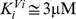); (vi) nucleotide dependence of trapping at µM levels. All of these values are the same order of magnitude as those reported in the literature for verapamil-activated Pgp (**Table 1**).

However, this model could not account for either the mixed-type inhibition exhibited by P_i_, or for the observed ATP dependence of its protective effect on V_i_ trapping [14], [23]. Analysis of the steady-state expression in this model (Eq. 1) revealed that 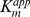 and 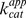 can be described compactly according to
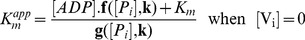
(23)

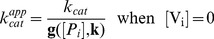
(24)


where **f** and **g** are functions of [*P_i_*] and the vector **k**. Thus, in the absence of ADP, the ratio between both parameters at any P_i_ concentration would be constant. However, in the presence of ADP in the reaction medium, the numerator of Eq. 23 is not reduced to *K_m_*, so the slope of the double-reciprocal plot is dependent on inhibitor concentration, a characteristic of mixed-type inhibition, as reported by Urbatsch et al. [23]. However, the explanation for the inhibition they observed is highly unlikely to be ADP accumulation following hydrolysis, since Pgp has a low catalytic rate, and the ATP concentration was kept constant during the experiment by a regenerating system.

Analysis of trapping with ATP/ADP uncovered another discrepancy between the output of the modeled *Elemental Cycle* and experimental evidence. According to Eqs. 11 and 12, at saturating V_i_ concentration the *IC*_50_ values of both nucleotides are defined by
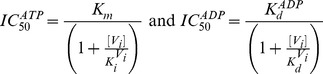



Considering that (i) the numerators follows the relationship 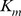
>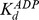 and (ii) 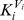
is always > 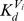, since 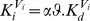 (Eq. 4d) and 
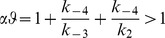
 (Eq. 5) for any value of the rate constants, the model cannot reproduce the experimental observation that 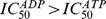
for any V_i_ concentration. To match the reported data 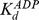 would need to be 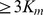. Additionally, the steeper concentration dependence reported experimentally [23] obviously reflects the binding of two nucleotides in the full catalytic cycle, and is in contrast with the Hill number of 1 obtained from the *Elemental Cycle*.

The relationship between P_i_ and V_i_ also revealed an additional element that makes the *Elemental Cycle* unsatisfactory. By simultaneously setting primary properties, such as 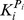 and 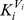
from ATP hydrolysis and *IC*_50_ for trapping with both ATP and ADP, for a given set of other fundamental properties (especially 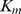 and 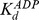), it was impossible to mimic the reported relationship between these two oxoanions. The 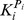 values for trapping with ADP and ATP reported by the simulation were half of the values obtained experimentally. These values could not be matched without changing the other reported properties, that is by either (i) increasing the competitive capacity of P_i_ (decreasing 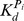); this change increases the P_i_ dependence of ATPase activity (i.e. by decreasing 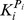 for activity), or (ii) decreasing the competitive capacity of V_i_ (increasing 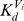); this change affects the 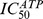 and 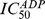 for trapping.

### The Temporal Behavior of the Elemental Cycle

Several considerations indicate that the observed slow kinetics of ADP trapping cannot be used to estimate the rate of ADP binding, as suggested by Urbatsch et al. [23], since this would yield 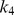  =  1.2×10^−5^ µM^−1^s^−1^. Such a low value for 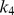, for the given 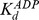 = 500 µM, would make ADP dissociation the rate-limiting step for ATP hydrolysis, even if 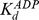 is as high as 1.5 M. In this regard, decreasing 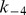 below 0.04 s^−1^ (keeping the other rates constant) has a profound effect on the catalytic cycle, decreasing the turnover rate and 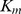 to unacceptable values. There is now ample consensus that catalysis is rate-limited in a concerted way, that is to say, there is no particular limiting step [18], [35]. This can be rationalized if 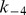 is quite similar to 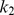, as long as the P_i_ dissociation rate is large (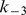 >> 1 s^−1^), a requirement that is fulfilled due to the low affinity of P_i_ for *E^ADP^* (and *F_ADP_*). Thus, the steady-state turnover rate would be limited only for the steady-state [*E^ATP^*] and [*F_ATP_*], which are in turn dependent on [*ATP*].

### The Temporal Behavior of the Alternating Cycle

In the case of ADP binding, it is not possible to incorporate additional unimolecular steps into the *Alternating Cycle* (as previously suggested [23]) without either affecting the overall 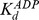 while preserving the effective forward rate, or affecting the overall forward rate while preserving the overall 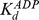. Nevertheless, Urbatsch et al. [32] considered fast binding of ADP followed by slow isomerisation but, again, *inside* the normal ATPase pathway. Our proposal on this issue, incorporated in the *Extended Alternating Cycle*, came from considering an alternative pathway for ADP binding (see the red reactions in **Figure 2**) *outside* the regular hydrolysis pathway. Thus, for V_i_ trapping, by either the fast pathway using ATP or the slower pathway using ADP, the final intermediates are the same, 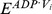 and 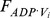. This is the case since for the ATP pathway, the equilibrium 
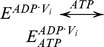
 (and the *F*-form equivalent) is almost completely shifted toward the left (i.e. *k*_1a_[*ATP*]/*k*_−1a_ <<1). Moreover, when the pulse of V_i_ and ATP is off (i.e. when ligand is removed), a rapid transition takes place toward the one-nucleotide trapped species (with 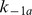 = 1 s^−1^). Thus, 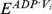 and 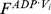 would be the trapped species present in the gel filtration column eluate, as observed experimentally [23].

Both one- and two-nucleotide trapped species have been captured for hamster Pgp, depending on the nucleotide analog and inhibitor employed. In the presence of fluoroaluminate (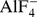), Sankaran et al. [36] identified two nucleotides as trapped/bound (ADP/ATP or 8-azido-ADP/8-azido-ATP) when Pgp was incubated with ATP or 8-azido-ATP. In contrast, only ADP/8-azido-ADP was trapped in the presence of V_i_. Beryllium fluoride (BeF_x_) behaves similarly to V_i_ in combination with nucleotides/8-azido-nucleotides [37]. Thus, the geometry of the transition state, dictated by the divalent cation, the inhibitor and the nucleotide analog used, determines the properties of the NBDs and the interaction between them, (i.e. 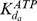).

The inclusion of an independent pathway for ADP binding thus offers important advantages in explaining the cycle of catalysis and trapping. However, a complete reaction pathway should, in principle, consider sequential binding of two ADP molecules, as in 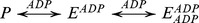 (and the *F*-form equivalent), in the same way that the *PE Alternating Cycle* proposes binding of two ATP molecules. Indeed, Pgp can bind two ADP (one in each NBD) in the absence of ATP. Qu et al. [38] reported the binding of two TNP-ADP molecules to Pgp by fluorescence titration, and Tombline et al. [34] found a Hill number of 1.7 for ADP binding to Pgp catalytic mutants. In addition, the ADP dependence of V_i_ trapping displayed cooperativity [23]. However, the species 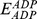 and 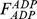 can only be formed by incubation of Pgp with ADP alone, and thus they will not exist during the physiological catalytic cycle. Thus, the existence of distinct trapped species depending on the nucleotide used might account for the differential sensitivity to collisional quenching observed for Pgp trapped with ATP compared to ADP [39].

In our simulation, the unusually small values assigned to the rate constants for the trapping pathways (i.e. *k*_±5a_ and *k*_1a_; **Table 4**) should be noted: association rate constants for ligand-enzyme interactions are normally in the range 10^−3^-10^3^ µM^−1^s^−1^. However, these values were set in order to reproduce, within the minimal reaction scheme, the kinetic behavior exhibited during trapping and release experiments. For example, consider V_i_ release in the presence of ATP by the path 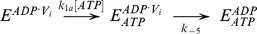; the outcome is that the ATP dissociation constant was effectively set to a high value, 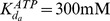. The setting of *k*_1a_ to a low value (the rate-limiting step) was due to the setting of *k*_−5_ to a high value, which was in turn based on kinetic analysis of the *Elemental Cycle*. As mentioned above, it is feasible to include additional steps in V_i_ release, 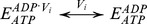, to allow assigning more reasonable values to these rate constants. The *k*_5a_ step, which explains slow trapping by ADP, can also be split into several conformational steps. Even ADP association with the bare enzyme can be slow due to the absence of constraints imposed by 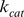 in the regular catalytic pathway. In this regard, we found up to five transitions in TNP-ADP binding to Pgp under pseudo-first order conditions, with the observed time constants spanning 5 orders of magnitude, ranging from ms to tens of seconds [40].

### The Concept of Alternating Catalysis

Alternating catalysis, which was originally proposed by Senior et al. [25], integrated two *Elemental Cycles* in tandem. It arises because of a mutual interaction between the two Pgp halves that allows only one NBD to be active for a particular protein conformation (i.e. NBD1 for *E*, NBD2 for *F*). Consequently, because binding of a second ATP to NBD2 is required to enable NBD1 to carry out hydrolysis (and vice versa), catalysis alternates between two *Elemental Cycles*. This characteristic is the crucial distinction between this mechanism and the *Sequential Mechanism* proposed by Sauna and Ambudkar [30], where alternation of the two *Elemental Cycles* has its origin in the nature of the ATP binding step, rather than the hydrolytic step. Thus, for the latter model, the presence of ATP bound at a particular NBD is proposed to prevent binding of a second ATP at the other NBD. Biochemical and structural evidence supports the existence of a ternary Pgp complex with two nucleotides bound; the currently accepted model of catalysis is that each NBD carries out the catalytic cycle in turn, enabled by the complementary NBD with ATP bound.

In the *Alternating Cycle*, during steady-state activity of the enzyme, at least one molecule of ATP is always bound (see **Figure 2**, grey cycle); each ATP molecule to be hydrolysed must bind to a previously formed Pgp⋅ATP complex. However, for a newly synthesized Pgp molecule in the cell, or at the beginning of an *in vitro* ATPase assay, the protein does not have any bound nucleotide. Thus, the priming reaction 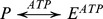 (and its equivalent for the *F*-form) must necessarily occur. This step has possibly been ignored in the past because it is “obvious”, but it is necessary to include it explicitly to provide a pathway for the protein to enter the catalytic cycle. As discussed below, this additional binding reaction generates changes in the ATP dependence of any measured variable, and suggests some new concepts about the catalytic mechanism. The simple *Elemental Cycle* simulation obviously cannot report interaction in the nucleotide dependence of any variable for the intact Pgp, since only one nucleotide is involved in the cycle. However, for the *Alternating Cycle*, the observation of *n*>1 for *in vitro* trapping with ATP arises because of nucleotide priming reactions (in the case of *in vitro* trapping with ADP, the simulation still reported *n* = 1, since only one ADP binding event was considered in this case). When simulating the *PE Alternating Cycle*, the value of the Hill number obtained for trapping is dependent on the ratio between the two ATP affinities and the type of coupling between the NBDs. Thus, for sequential binding of two ATP molecules, the Hill number ranges from: (i) *n* = 2, when the catalytic sites present no binding interaction (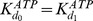) but show interdependence at the hydrolysis step (alternating catalysis, mutual exclusion of hydrolytic activity); (ii) 1<*n*<2, for a negative binding interaction (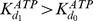) with again, inter-dependence of hydrolysis (e.g. alternating catalysis); and (iii) *n* = 1, for mutual exclusion in the binding (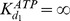; i.e. after binding of the first ATP, binding of a second ATP cannot occur) and independent hydrolysis; which is the case for either uncoupled/isolated half-molecules (the *Elemental Cycle*) or the *Sequential Mechanism* (*Elemental Cycles* in tandem). For Pgp undergoing a complete catalytic cycle at both NBDs, as already discussed, option (iii) is discarded. However, due to the absence of any quantitative reports of the value of *n* for wild-type hamster Pgp, it is not possible to rule out either of the first two possibilities based on trapping experiments. However, several pieces of evidence point towards the second option (a negative binding interaction):

- The *Sequential Mechanism* [30] proposed allosteric control of the ATP binding affinities of the two NBDs. In this model, the alternating feature of the hydrolysis arises from the impossibility of a two-nucleotide species due to dramatic reduction in the binding affinity for a second nucleotide when one is already bound. Evidence was presented that correlated decreased affinity for drug with decreased affinity for nucleotide, thus, accounting for the release of both at the end of the catalytic cycle. Viewing this proposal using our model, the species 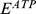 must have low ADP affinity in the empty NBD (i.e. NBD2) to account for release of ADP from 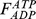 (see **Figure 2**). The low value of 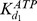 could be explained by extending that property to ATP. The *Sequential Mechanism* is, therefore, an extreme case of a negative binding interaction (interaction factor →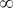). In our case, modeling the *PE Alternating Cycle* with an interaction factor of 200 (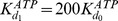) yielded *n* = 1.25 for V_i_ trapping with ATP and preserved the other properties of the catalysis and trapping.
- The observed Michaelis-Menten (*n*≅1) behavior of the ATP dependence of hydrolysis requires either complete independence (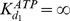; no binding of a second ATP) or a higher priming reaction affinity (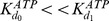). Although high affinity binding of ATP has not been reported, this is not conclusive, since the various reports are imprecise or incomplete. Plots of the nucleotide dependence of ATP hydrolysis by Pgp have often started from a relatively high nucleotide concentration (e.g. 50 µM), thus missing details of the low concentration part of the curve. It should be noted that the inclusion of a high affinity priming reaction generates curves for ATP dependence that deviate only very slightly from the single-binding model, so that it would only be perceptible in either log or log-log plots. In addition, the low concentration part of the curve could only be taken into account using a weighted fitting to a Hill model; a non-weighted simple Michaelis-Menten fitting would miss the high affinity component.
- An interesting report by Buxbaum [33], which measured hydrolysis of ATP in the µM range, reported significant deviation from hyperbolic behavior. Upward curvature in the log-log plot was observed at low ATP concentrations, with a breakpoint at ∼10 µM, which can only be explained by interaction between the NBDs during catalysis. In addition, the author reported that activation of ATP hydrolysis by verapamil occurred only at high ATP concentration, which might be reconciled with our model by adding a *priming cycle* for ATP hydrolysis (i.e. hydrolysis of the one-nucleotide species) uncoupled from drug transport.

**Figure 12:**
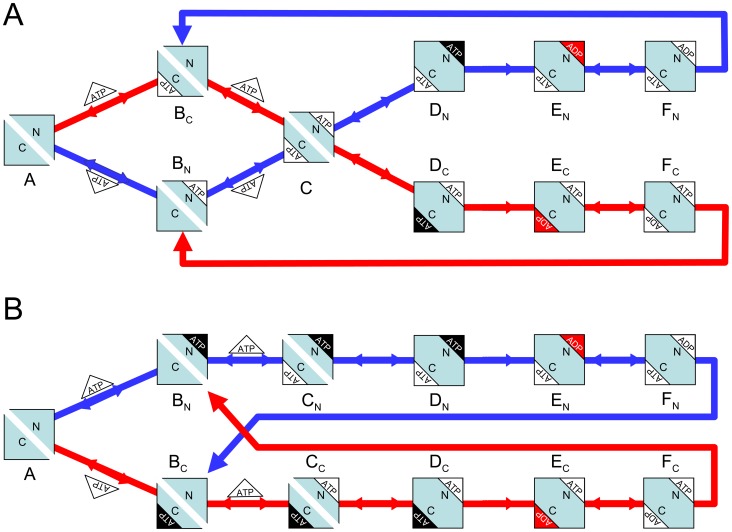
Cartoons depicting the *Alternating Cycle*. (A) Random binding model adapted from Urbatsch et al. [32]. (B) Sequential binding model proposed in this work (see **Figure 2**). White triangles represent ATP, black triangles represent ATP committed for hydrolysis; ADP·P_i_ is shown in red, ADP in white. The subscripts of the intermediates (A to F) correspond to the N and C terminal halves of the protein. Closure of the NBD dimer interface is reflected in the fusion of both halves of the protein square. Flow through each half-cycle is represented by the blue and red arrows.

The essential steps in the alternating mechanism proposed by Urbatsch et al. [32] are depicted in the cartoon in **Figure 12A**. The ATP binding reaction is conceived as a random process, producing the two-nucleotide intermediate (C) without any distinction in their binding affinities. Subsequently, this intermediate chooses a pathway toward either D_N_ or D_C_, depending on which NBD last hydrolyzed ATP. This model requires the intermediate C to have some type of “memory”, i.e. C must possess some intrinsic difference based on the last hydrolytic event, for example, a slight difference in the forward rate (C→D) between NBD1 (N-end) and NBD2 (C-end). However, by definition, C must be identical regardless of the branch used for the priming binding step, so that the next step would have to be randomly selected. This places Senior's *Alternating Mechanism* in an awkward position: in the forward step from C, there is no guarantee of alternation of the two half-cycles.

In contrast, our proposal for the *Alternating Cycle* (**Figure 12B**) considers sequential ATP binding with decreased affinity for the second nucleotide, to produce distinct two-nucleotide intermediates, C_N_ and C_C_. In this model, alternation is guaranteed since there is no common intermediate; there is no need to propose the existence of memory for any species. The model in **Figure 12B** is equivalent to that shown in **Figure 2**, where one branch (blue) corresponds to the *E*-form of Pgp, and the other (red) to the *F*-form, and the intermediate A corresponds to the *P* form. Thus, the release of ADP and the transition between kinetics forms in **Figure 2** (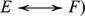, are represented by the transitions F_N_ 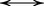
B_C_ and F_C_ 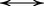
B_N_ in **Figure 12B**.

In summary, in our implementation of the *Alternating Cycle* in **Figure 12B**, the needed asymmetry for the alternation of the two paths is structural in origin (it is contained within the overall cycle), and arises from the reciprocal negative allosteric interaction between the NBDs. On the other hand, Senior's *Alternating Cycle* shown in **Figure 12A** is functional in origin (it is facilitated by the functioning of the cycle), since it depends on the “memory” of a particular intermediate for the previous hydrolytic event. This feature ultimately arises because the model does not consider the priming reactions, and considers only the cycling part of the scheme.

### The Occluded State

The concept of occlusion proposed by Tombline and Senior [41] can be easily supported in our current model, as depicted in **Figure 12B** by the transitions 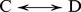. For this, the conformational transition between the non-occluded (C) and occluded (D) two-nucleotide species would be represented by 
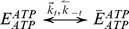
 in the kinetic schemes, where *E* denotes the non-occluded state and 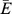 the occluded state, with equilibrium constant *K_t_*. This transition is not a binding event, since there is no direct exchange (association or dissociation) of ATP; so that the apparent ATP affinity of the occluded species (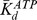), would, in fact, be the overall ATP dissociation constant for the second nucleotide, as represented by the serial equilibria, 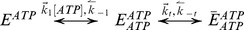, with 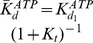. Thus, as occlusion progresses in the forward direction (*K_t_*>>1), the apparent ATP binding affinity is significantly increased relative to the true “microscopic” binding affinity (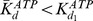). However, this additional transitional step is not necessary to account for the experimental data reported with Pgp mutants and ATP analogs, as explained below.

According to our interpretation, the work of Tombline et al. with Pgp mutants [34] might correspond to a pseudo-equilibrium binding titration of the bare enzyme, due to impairment in the hydrolytic rate constant, which reduced 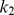 by a factor of 1000. **Figure S2A** shows the steady-state distribution at various ATP concentrations of the intermediates 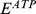 and 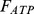, which closely matches the equilibrium 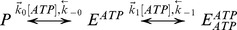 (and the *F*-form equivalent). By decreasing both rate constants of the priming reaction (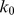 and 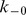, keeping 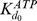 constant) the experimental data of Tombline et al. [34] could be simulated. After removal of free ligands, the reactions that describe the system, 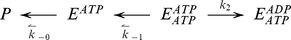 (and the *F*-form equivalent) predict occlusion of the nucleotide (equivalent to trapping without V_i_). This arises mainly from formation of the species 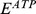 (and 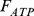) due to slower conversion of 
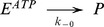
, and also increased formation by dissociation of two-nucleotide species 
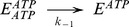
(and the *F*-form equivalent), which built up to a higher steady-state concentration because of greatly reduced hydrolysis. We decreased 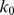 and 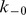 by 1000-fold for the Pgp mutants and assumed that after passage through a gel filtration column (∼30 s) almost all of the two-nucleotide species become one-nucleotide species (since 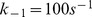, ∼2000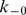). **Figure S2B** shows the fraction of Pgp with retained nucleotide (i.e. occluded species) at various ATP concentrations, and reports an overall affinity for ATP (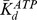) quite close to 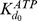, in the µM range. In addition, the variable effect of several drugs on the steady-state stoichiometry of occlusion at a fixed nucleotide concentration [27] might be accounted for by a differential effect on 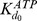 (see **Figure S3**).

We recently reported that the binding to Pgp of the poorly-hydrolysable analog ATPγS exhibits a biphasic isotherm, with 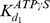 = 6 µM and 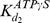 = 740 µM [29], with the highest affinity binding component assumed to correspond to the occluded state. However, our interpretation based on the current model is that 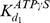 may correspond to the affinity of the bare enzyme (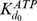, see **Figure S3**). This would account for the 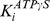
of 6 µM observed for the inhibition of the ATP hydrolysis by ATPγS [29], corresponding to competition with ATP for the high affinity site (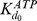) of bare Pgp.

In consequence, our model is compatible with the proposal of occlusion; but differs from it conceptually in the following way. The occluded state (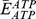) identified experimentally has a tightly bound ATP (µM affinity) committed to hydrolysis, while a second molecule is bound to the complementary NBD. In our interpretation, this intermediate (D_N/C_ in **Figure 12A**) corresponds to the species 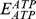 and 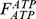 in **Figure 8**; it has one NBD with high affinity (e.g. NBD1 and NBD2 for the *E* and *F* isoforms, respectively), and is represented by intermediates C_N/C_ in **Figure 12B**. That is to say, in our model it is not necessary to include a conformational transition to the occluded state, since the high affinity site exhibited by this state actually corresponds to the site that bound the first nucleotide molecule to the bare enzyme. Consequently, occlusion would not necessarily reflect an increase in affinity of the NBD with ATP already bound, following binding of a second ATP in the complementary NBD. Rather, it might correspond to the conformational change that enables the high affinity NBD to hydrolyze the committed nucleotide, thus preparing the enzyme for the hydrolytic step. This could occur concurrently or after the binding of a second ATP, represented in **Figure 12B** as the transition 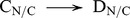. The occluded state is easily incorporated into the kinetic scheme in **Figure 2** as the transitions 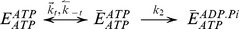, however, as indicated previously, from a kinetic point of view it is not necessary to include this feature in our model.

## Conclusions

The detailed analysis provided in this work underscores the fact that the mechanism underlying the kinetics of Pgp-mediated ATP hydrolysis must be much more complex than that proposed in previous models. Our goal was to incorporate the wealth of experimental data accumulated for hamster Pgp into a consistent kinetic simulation of the catalytic cycle. Implementation of the *Elemental Cycle* in the *Alternating Mechanism* (as originally proposed by Senior's group [25]) adequately explains (i) the time-domain and steady-state experimental data for ATP hydrolysis with respect to ATP, ADP and V_i_ concentrations; (ii) the steady-state experimental data for ATP/ADP dependence of V_i_ trapping; and (iii) the kinetics of V_i_ trapping with ATP. However, it fails to satisfactorily explain (a) the effect of P_i_ on ATPase activity; (b) the relationship between *IC*_50_ for ATP/ADP on V_i_-trapping; (c) cooperativity of ATP hydrolysis at low ATP concentrations; (d) the observed protective effect of P_i_ on V_i_-trapping with respect to the *IC*_50_ for ATP/ADP; (e) the steep concentration dependence observed for V_i_ trapping with ADP/ATP; (f) the kinetics observed for V_i_ trapping with ADP; (g) the kinetics observed for V_i_ release from the trapped-species; and (h) detection of species with only one trapped nucleotide. Development of the *Extended Alternating Cycle* allowed us to include additional kinetic steps to account for most of the deficiencies (c)-(h) of the original model (however, observations (a) and (b) still remain unexplained). **Figure 9** summarizes the ATP dependence of several biochemical variables in the *PE Alternating Cycle* of Pgp, according to the parameters given in **Tables 2** and **3**. This proposed model introduces both priming and trapping reactions into the kinetic scheme, and is able to account for the observed high affinity of Pgp for ATP without any reference to the occluded state, thus avoiding assigning special properties to any intermediate in the cycle. A new interpretation of the occlusion phenomenon also emerges from the model. Future work will be needed to model a comprehensive reaction scheme to explain the complete data-set of biochemical observations.

## Supporting Information

Figure S1
**Inhibitory effect of ADP on Pgp ATPase activity for the** ***Elemental Cycle*** **.** (A) 3D plot from the evaluation of 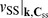 with 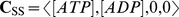, (B) Double-reciprocal plot from the evaluation 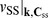 with 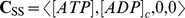 for [*ADP*]*_c_* = 0 (red), 250 (green), 500 (yellow) and 1000 µM (blue), with ATP concentrations ranging upwards from 10 µM. Values of **k** are given in **Table 2**.(TIF)

Figure S2
**ATP dependence of the concentration of several intermediates in the** ***PE Alternating Cycle*** **for catalytic mutants of Pgp.** Based on **Figure 2** and the values of **k** given in **Tables 2** and **3**, but substituting the following values: *k*_0_ = 0.01 µM^−1^s^−1^, *k_−_*_0_ = 0.05 s^−1^ and *k*_2_ = 0.02 s^−1^ (a 1000-fold impairment in the original rate constants). (A) Concentration of intermediates: [*P*] (blue), 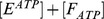 (red), and 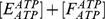 (green). (B) Fraction of Pgp with retained nucleotide, according to the function 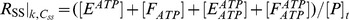 with 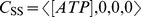. The synthetic data from the model (blue symbols) were fitted to a Hill equation (red line), yielding *K*_½_ = 5.1 µM and *n* = 1.01.(TIF)

Figure S3
**Effect of altering the affinity of the priming reaction on retention of nucleotides for catalytic mutants of Pgp.** 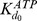 was altered by changing *k_−_*_0_, while imposing a 1000-fold impairment in the original rate constants (*k*_0_ = 0.01 µM^−1^s^−1^ and *k*_2_ = 0.02 s^−1^). The fraction of intermediates with retained nucleotide was evaluated by 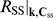 (as defined in **Figure S2**) with 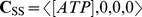, relative to the retained fraction for the original 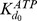  =  5 µM. Based on **Figure 2** and the values of **k** given in **Tables 2** and **3**.(TIF)

Figure S4
**Simulation of the stoichiometry of trapped ATPγS based on the** ***PE Alternating Cycle*** **.** ATP dependence of the stoichiometry of trapped nucleotide based on **Figure 2**, according to the function 
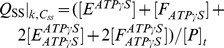
 with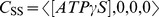, for values of **k** given in **Tables 2** and **3**, but considering *k*_0_ = 0.01 µM^−1^s^−1^, *k*_1_ = 1×10^−4^ µM^−1^s^−1^ and *k*_2_ = 0.02 s^−1^ (a 1000-fold impairment in the original values). The synthetic data from the model (blue symbols) were fitted to a two-site binding model (red line), yielding 
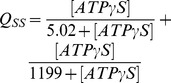
.(TIF)
