# Correction: Kinetic Validation of the Models for P-Glycoprotein ATP Hydrolysis and Vanadate-Induced Trapping. Proposal for Additional Steps

**DOI:** 10.1371/journal.pone.0101836

**Published:** 2014-06-26

**Authors:** The PLOS ONE Staff

There are errors in Equations 4, 5, 14b, and 15b. In Equations 4 and 5, phis were changed to thetas during the typesetting process; the publisher apologizes for the errors. In Equations 14b and 15b, “when [ADP]  =  0” should read “when [ATP]  =  0.” Please see the correct equations here.
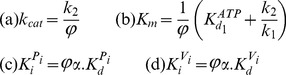
(4)




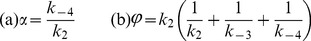
(5)




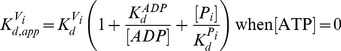
(14b)




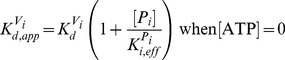
(15b)

