# Supplementary figures and images for: Kinetic Validation of the Models for P-Glycoprotein ATP Hydrolysis and Vanadate-Induced Trapping. Proposal for Additional Steps
Source: PLoS One. 2014 Jun 4;9(6):e98804. doi: 10.1371/journal.pone.0098804 (PMC4045855; doi:10.1371/journal.pone.0098804)

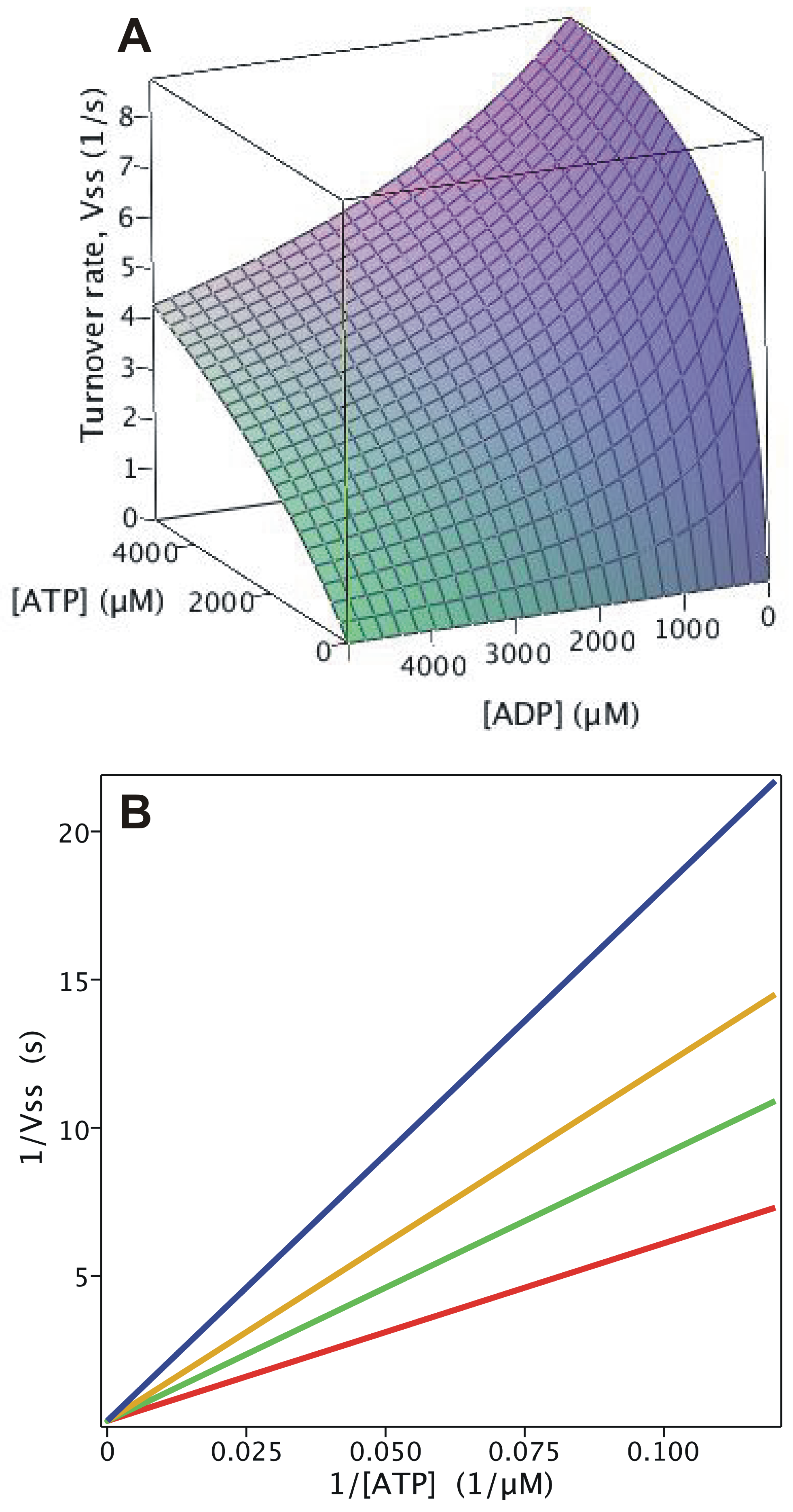

Supplement: Figure S1 — Inhibitory effect of ADP on Pgp ATPase activity for the Elemental Cycle . (A) 3D plot from the evaluation of with , (B) Double-reciprocal plot from the evaluation with for [ADP]c = 0 (red), 250 (green), 500 (yellow) and 1000 µM (blue), with ATP concentrations ranging upwards from 10 µM. Values of k are given in Table 2 . (TIF) [file pone.0098804.s001.tif]

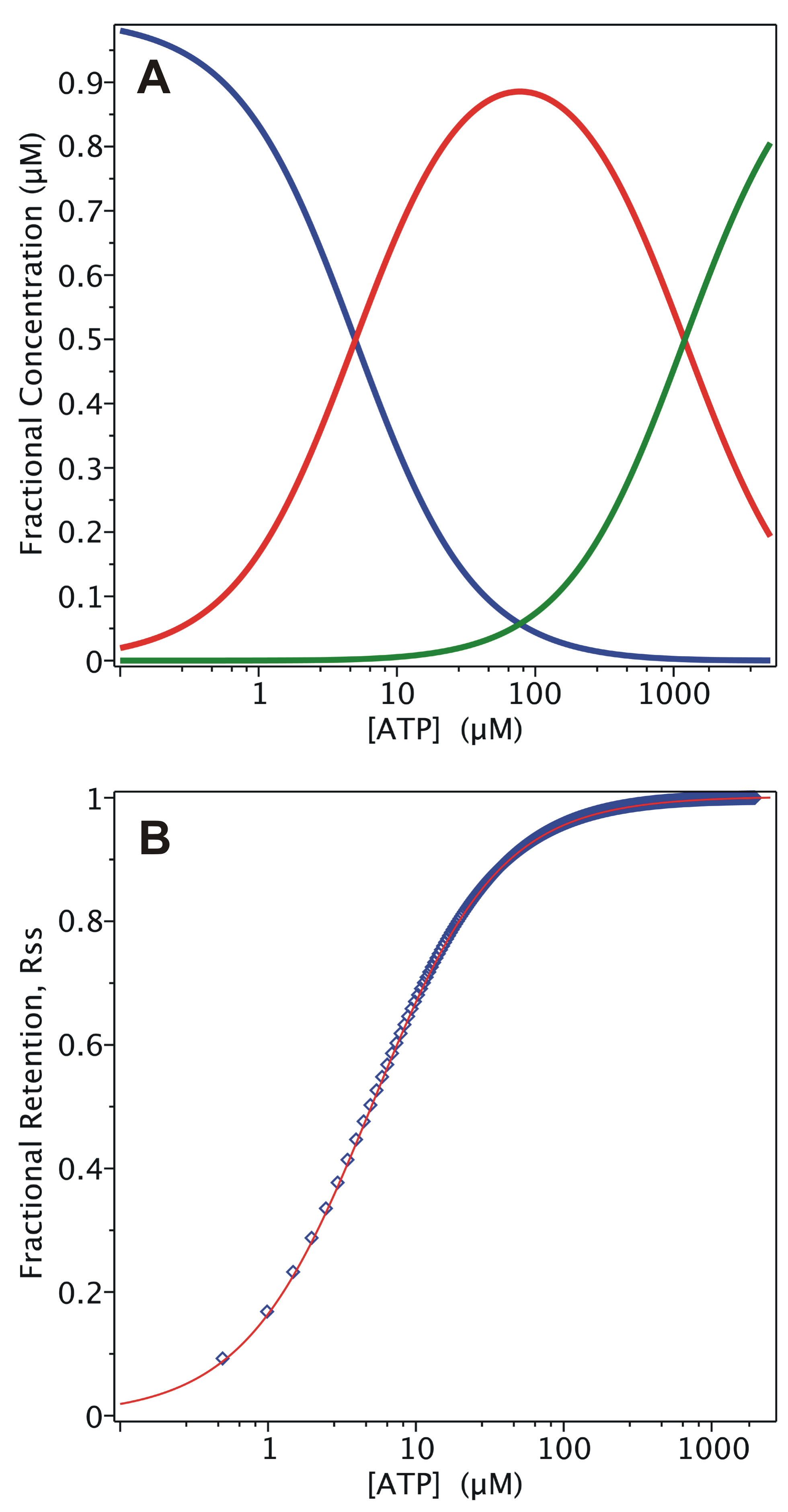

Supplement: Figure S2 — ATP dependence of the concentration of several intermediates in the PE Alternating Cycle for catalytic mutants of Pgp. Based on Figure 2 and the values of k given in Tables 2 and 3 , but substituting the following values: k 0 = 0.01 µM−1s−1, k− 0 = 0.05 s−1 and k 2 = 0.02 s−1 (a 1000-fold impairment in the original rate constants). (A) Concentration of intermediates: [P] (blue), (red), and (green). (B) Fraction of Pgp with retained nucleotide, according to the function with . The synthetic data from the model (blue symbols) were fitted to a Hill equation (red line), yielding K ½ = 5.1 µM and n = 1.01. (TIF) [file pone.0098804.s002.tif]

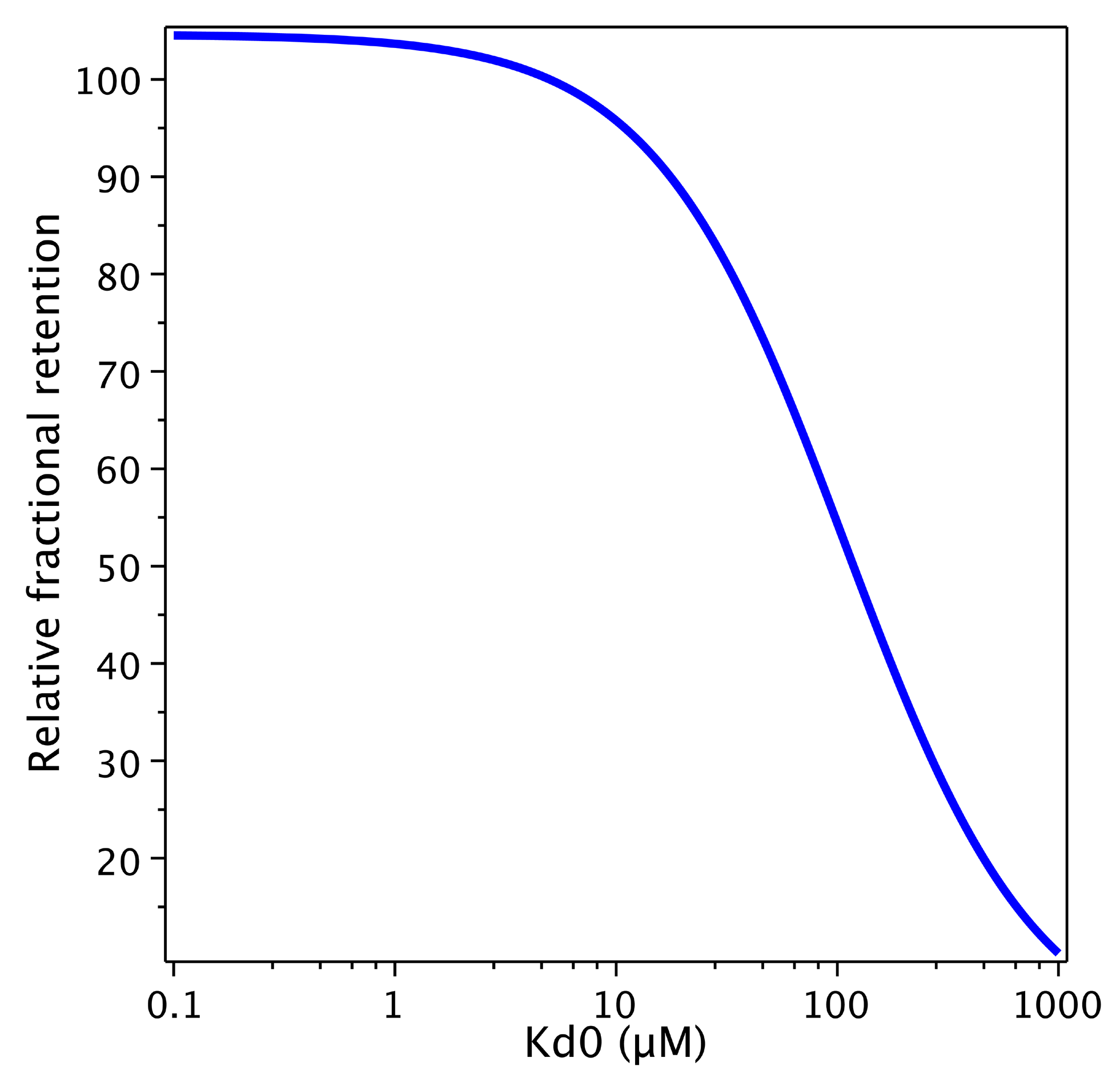

Supplement: Figure S3 — Effect of altering the affinity of the priming reaction on retention of nucleotides for catalytic mutants of Pgp. was altered by changing k− 0, while imposing a 1000-fold impairment in the original rate constants (k 0 = 0.01 µM−1s−1 and k 2 = 0.02 s−1). The fraction of intermediates with retained nucleotide was evaluated by (as defined in Figure S2) with , relative to the retained fraction for the original = 5 µM. Based on Figure 2 and the values of k given in Tables 2 and 3 . (TIF) [file pone.0098804.s003.tif]

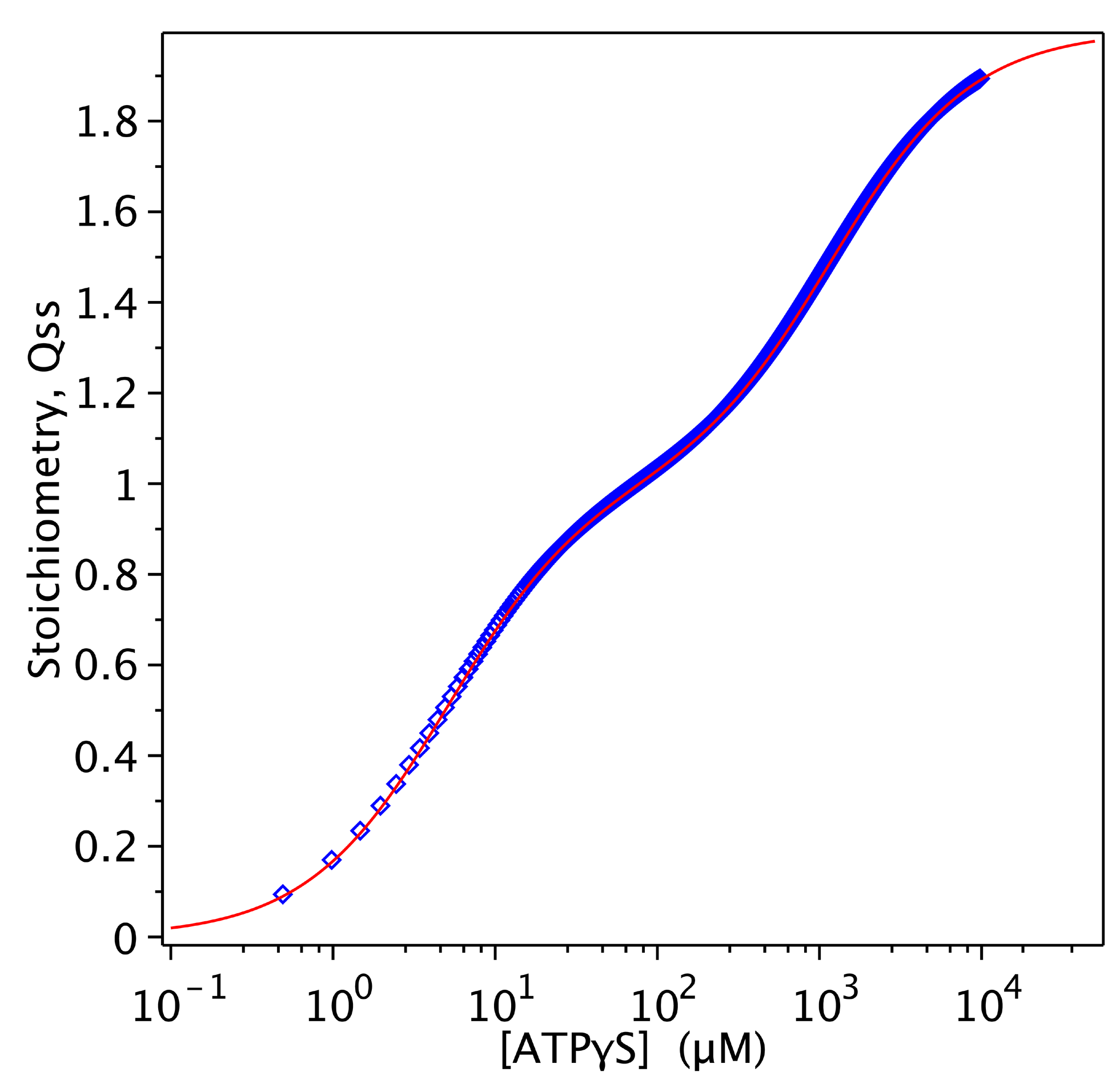

Supplement: Figure S4 — Simulation of the stoichiometry of trapped ATPγS based on the PE Alternating Cycle . ATP dependence of the stoichiometry of trapped nucleotide based on Figure 2 , according to the function with, for values of k given in Tables 2 and 3 , but considering k 0 = 0.01 µM−1s−1, k 1 = 1×10^−4^ µM−1s−1 and k 2 = 0.02 s−1 (a 1000-fold impairment in the original values). The synthetic data from the model (blue symbols) were fitted to a two-site binding model (red line), yielding . (TIF) [file pone.0098804.s004.tif]
